# Supplementary figures and images for: Multiple membrane extrusion sites drive megakaryocyte migration into bone marrow blood vessels
Source: Life Sci Alliance. 2018 May 22;1(2):e201800061. doi: 10.26508/lsa.201800061 (PMC6211653; doi:10.26508/lsa.201800061)

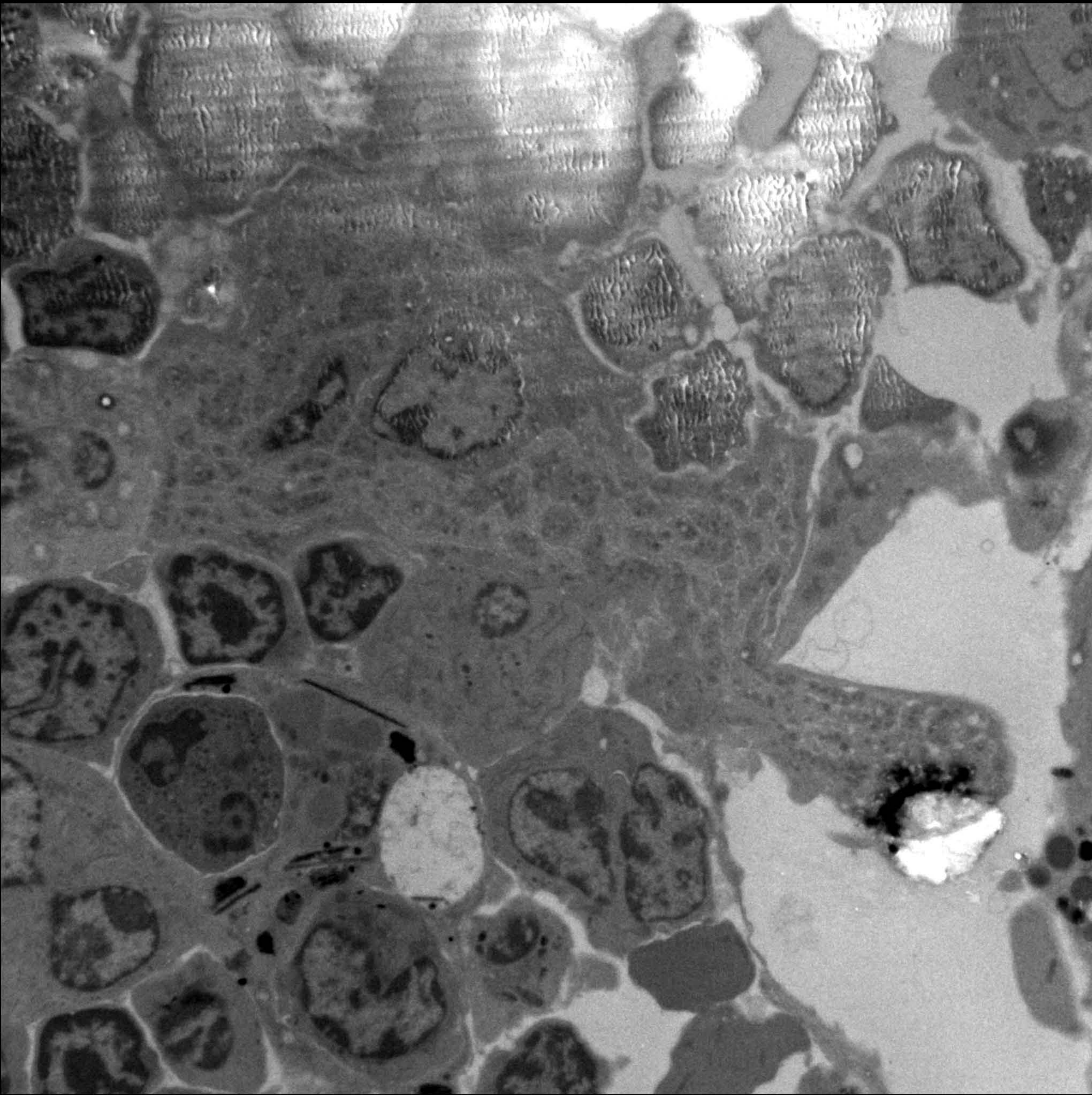

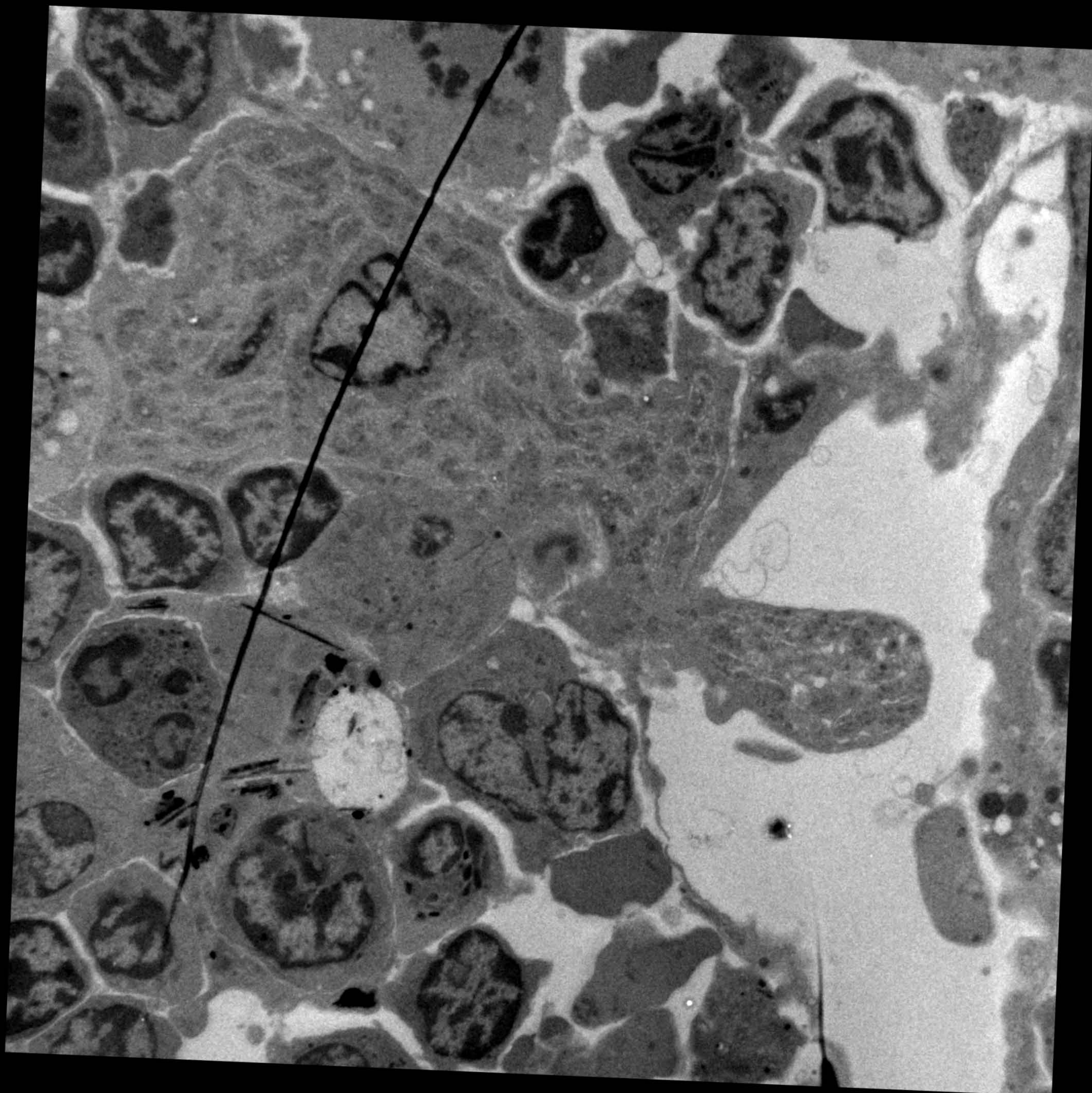

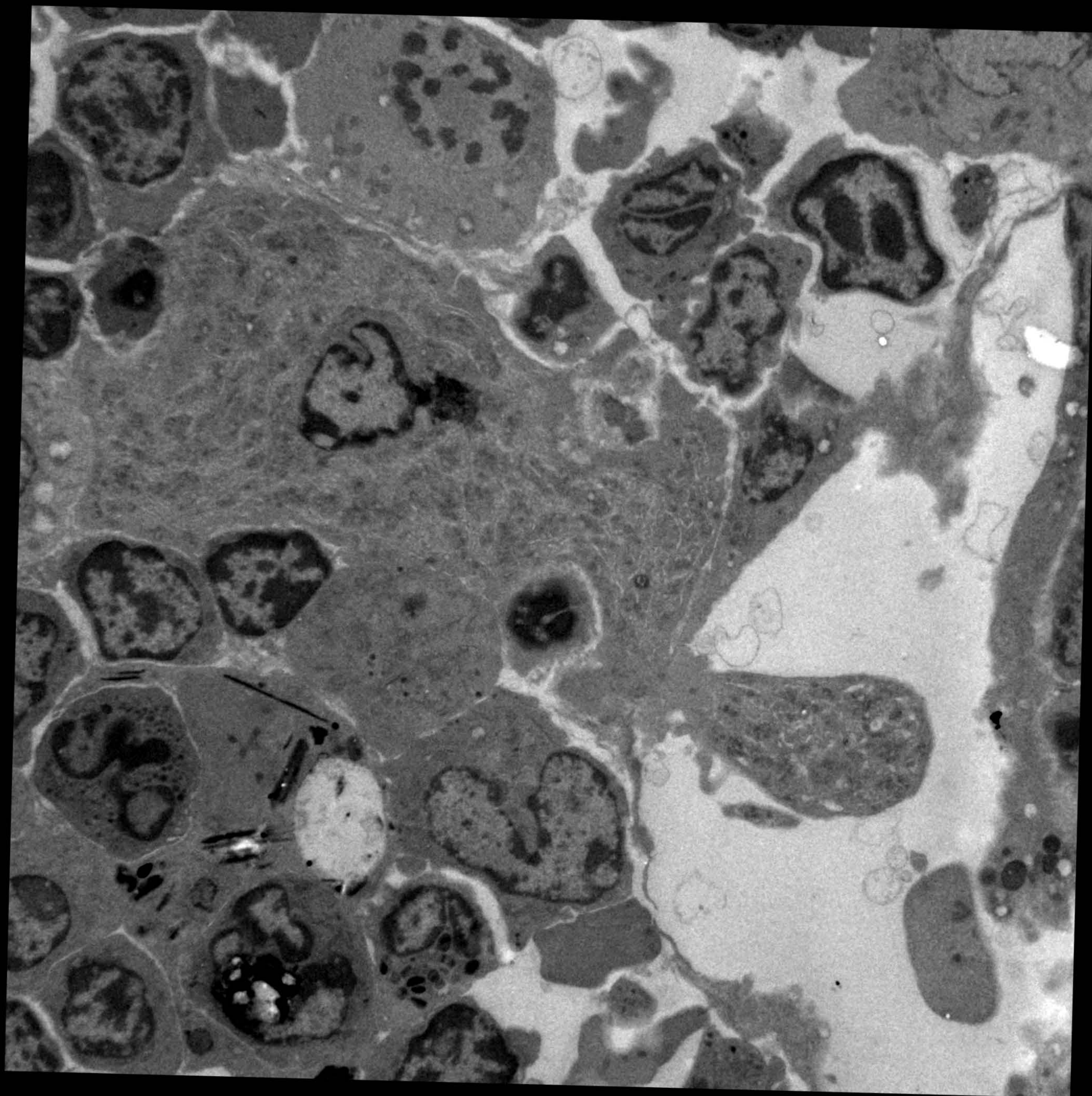

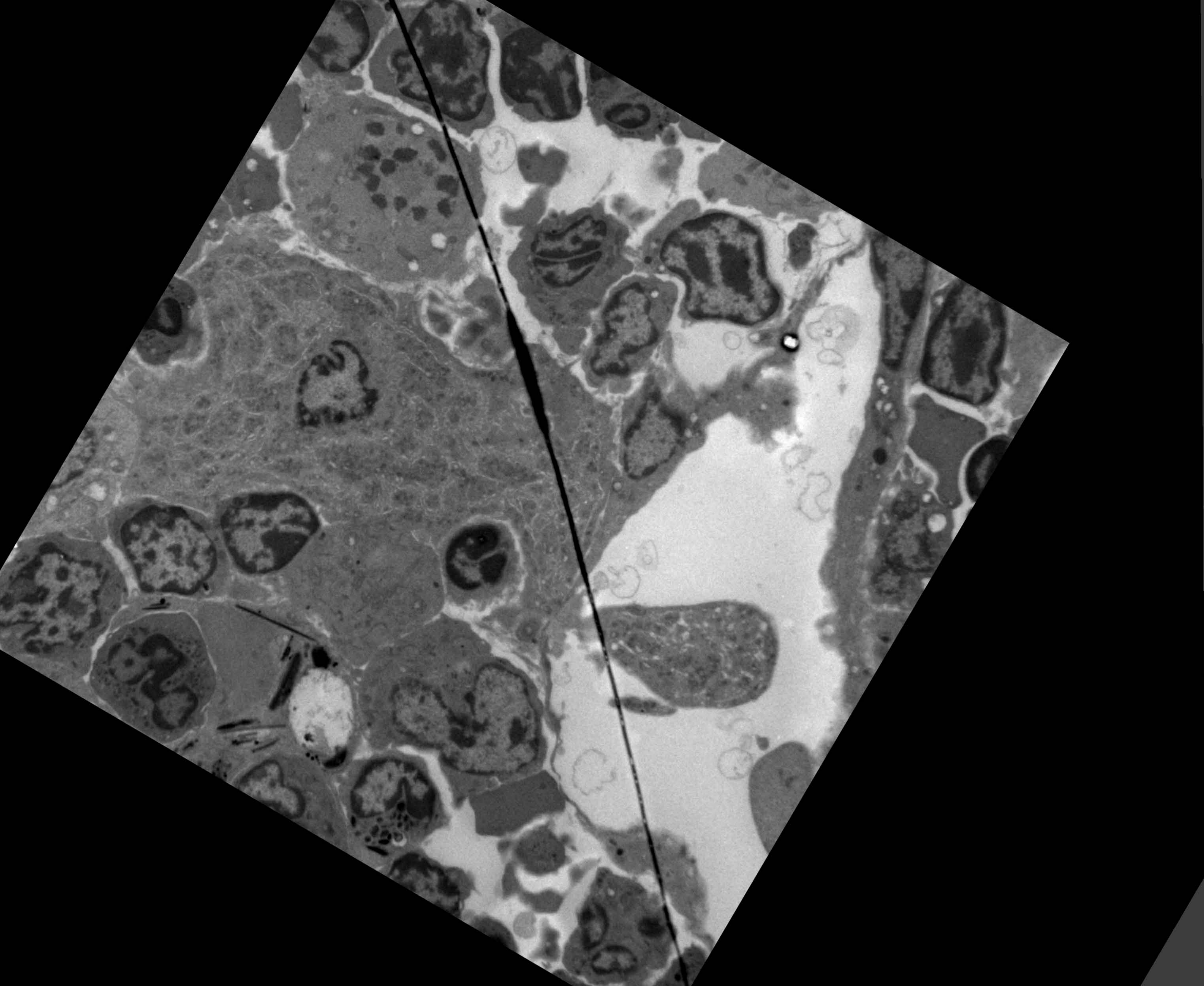

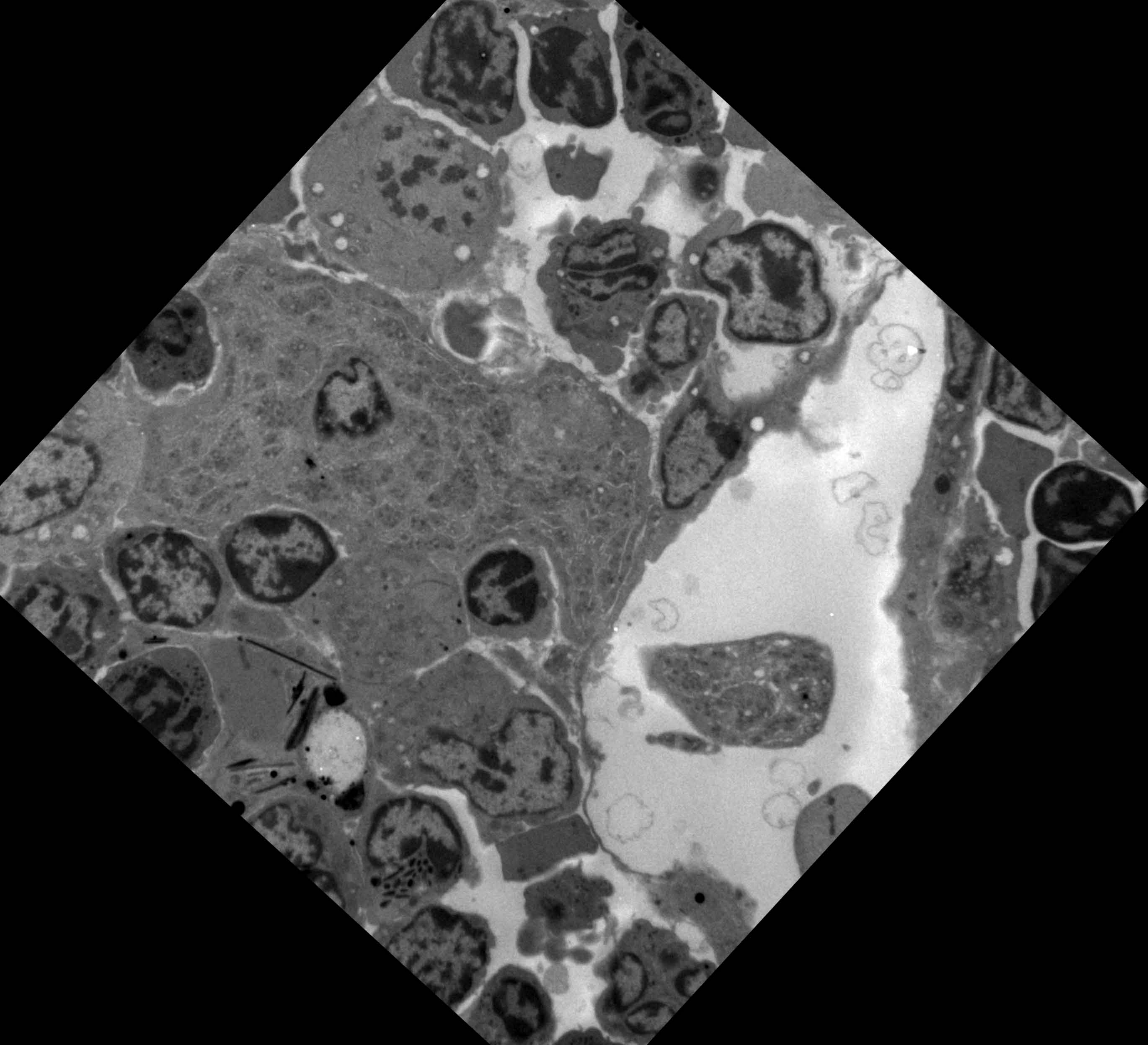

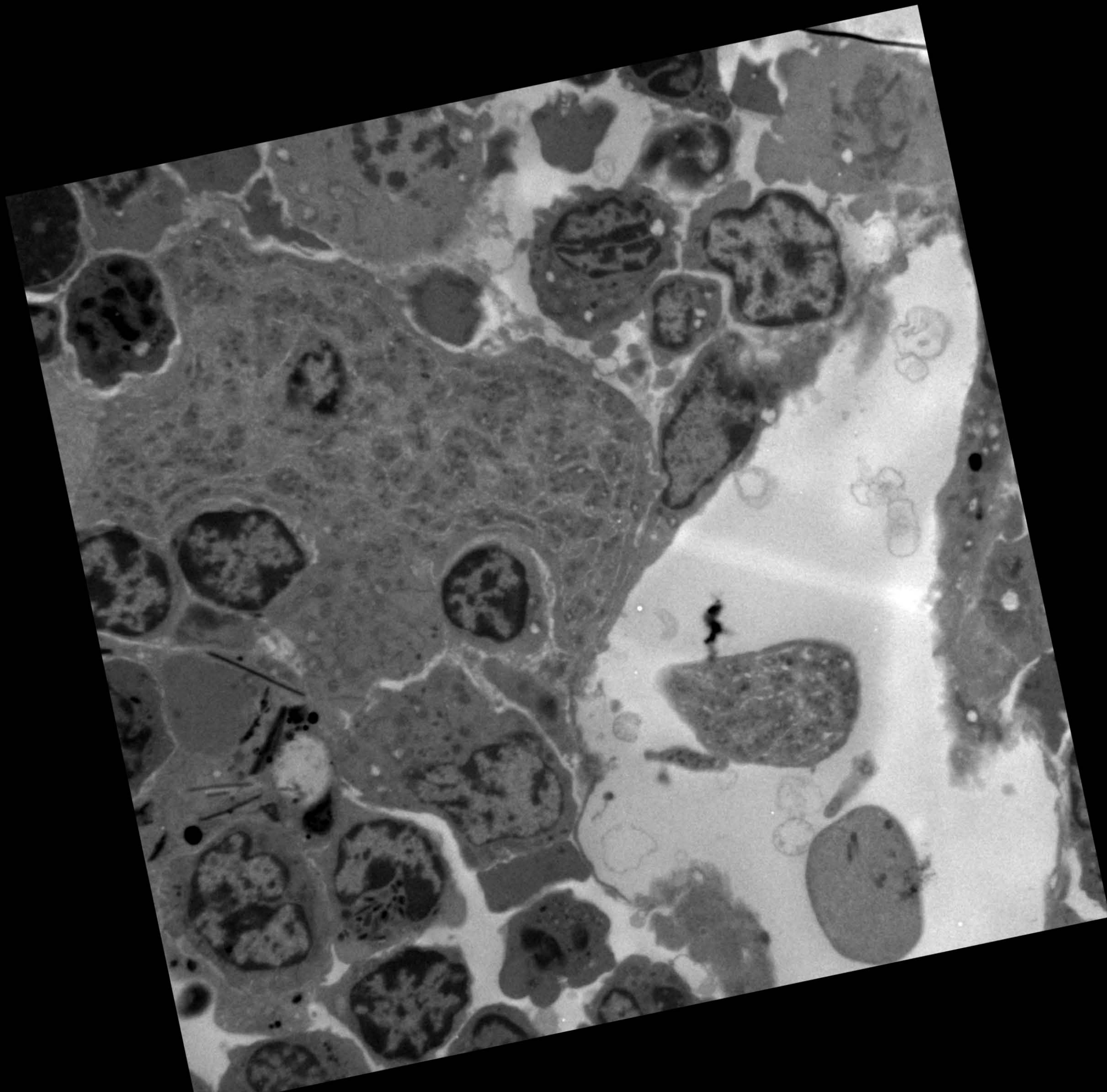

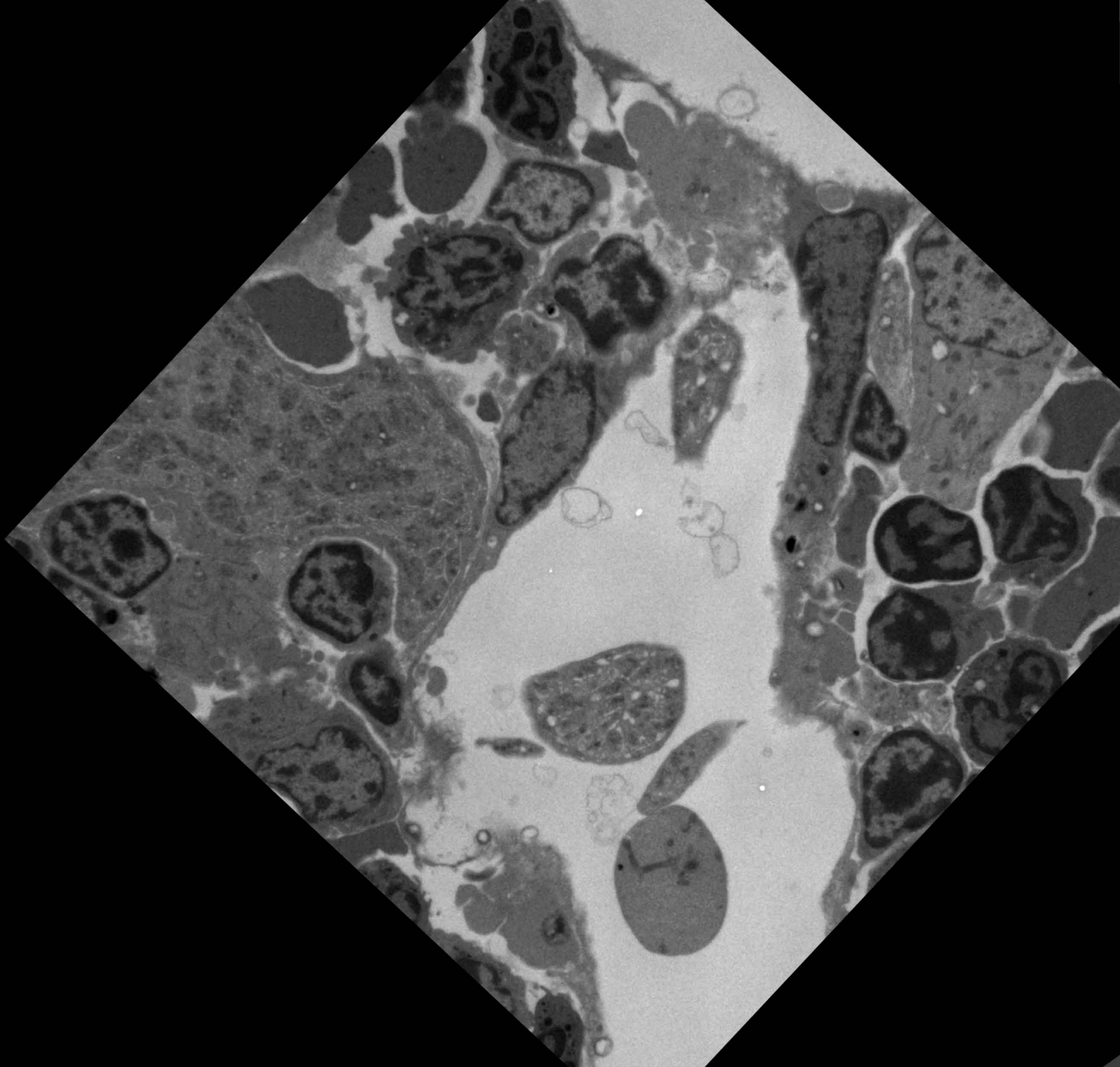

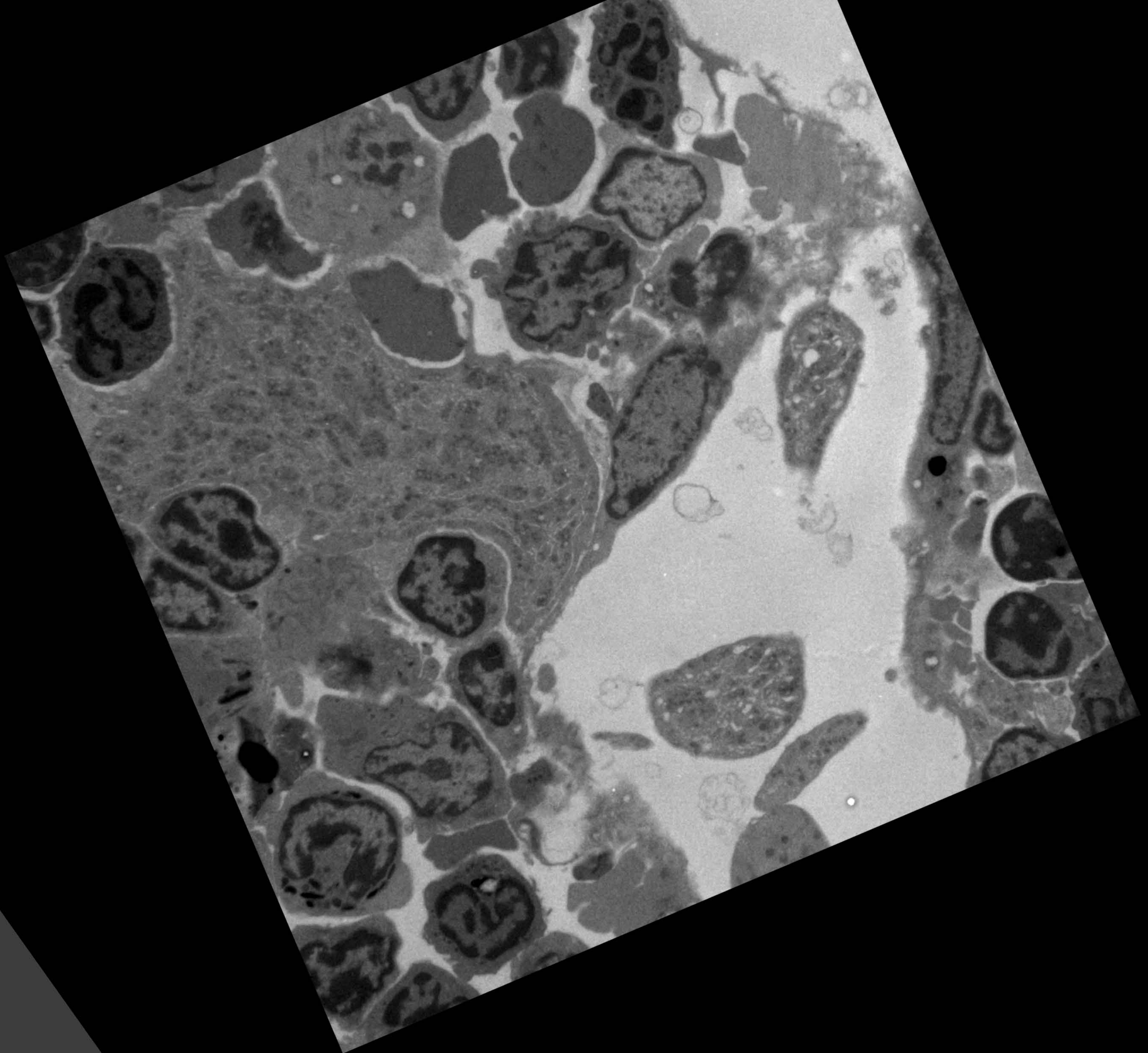

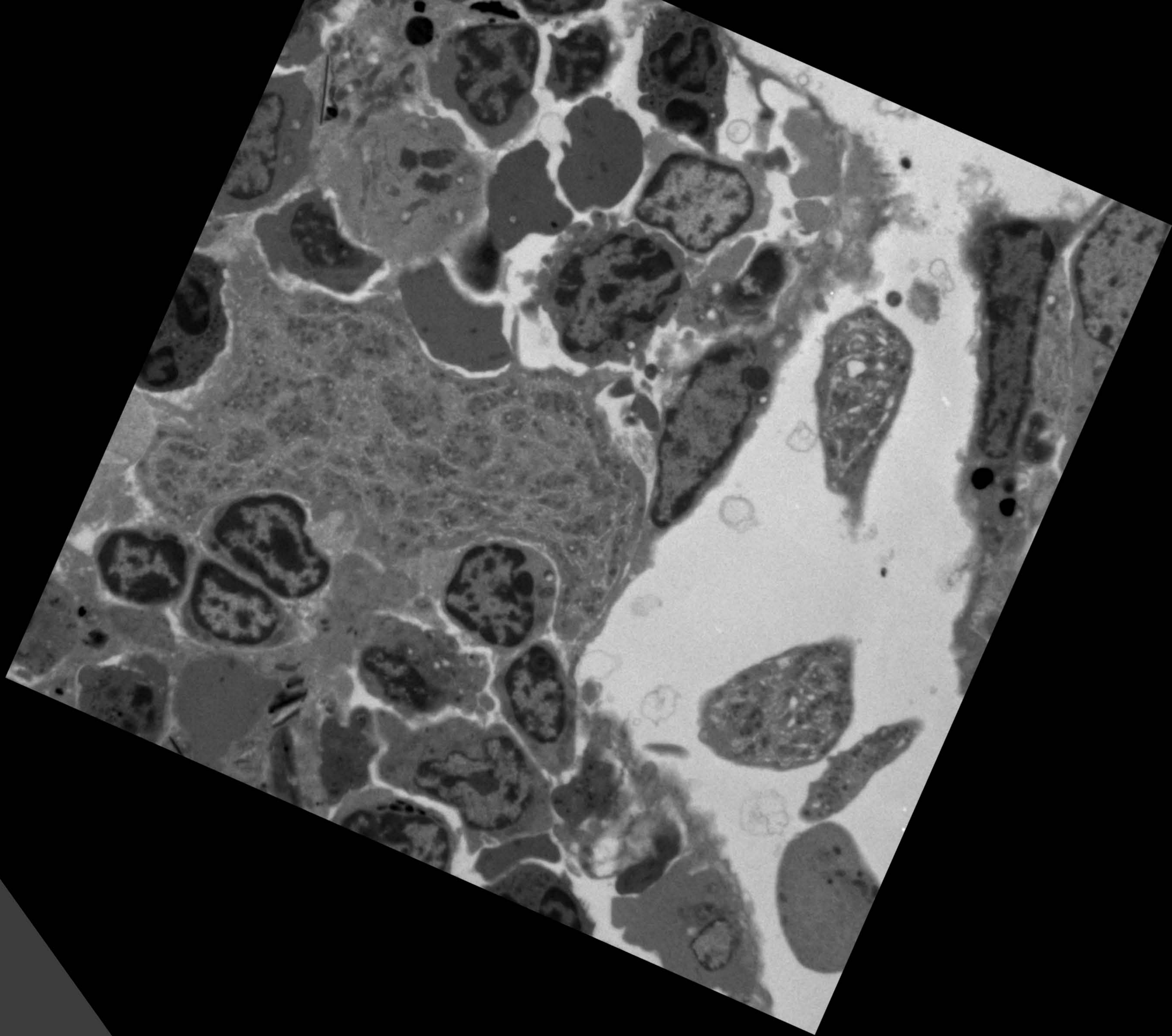

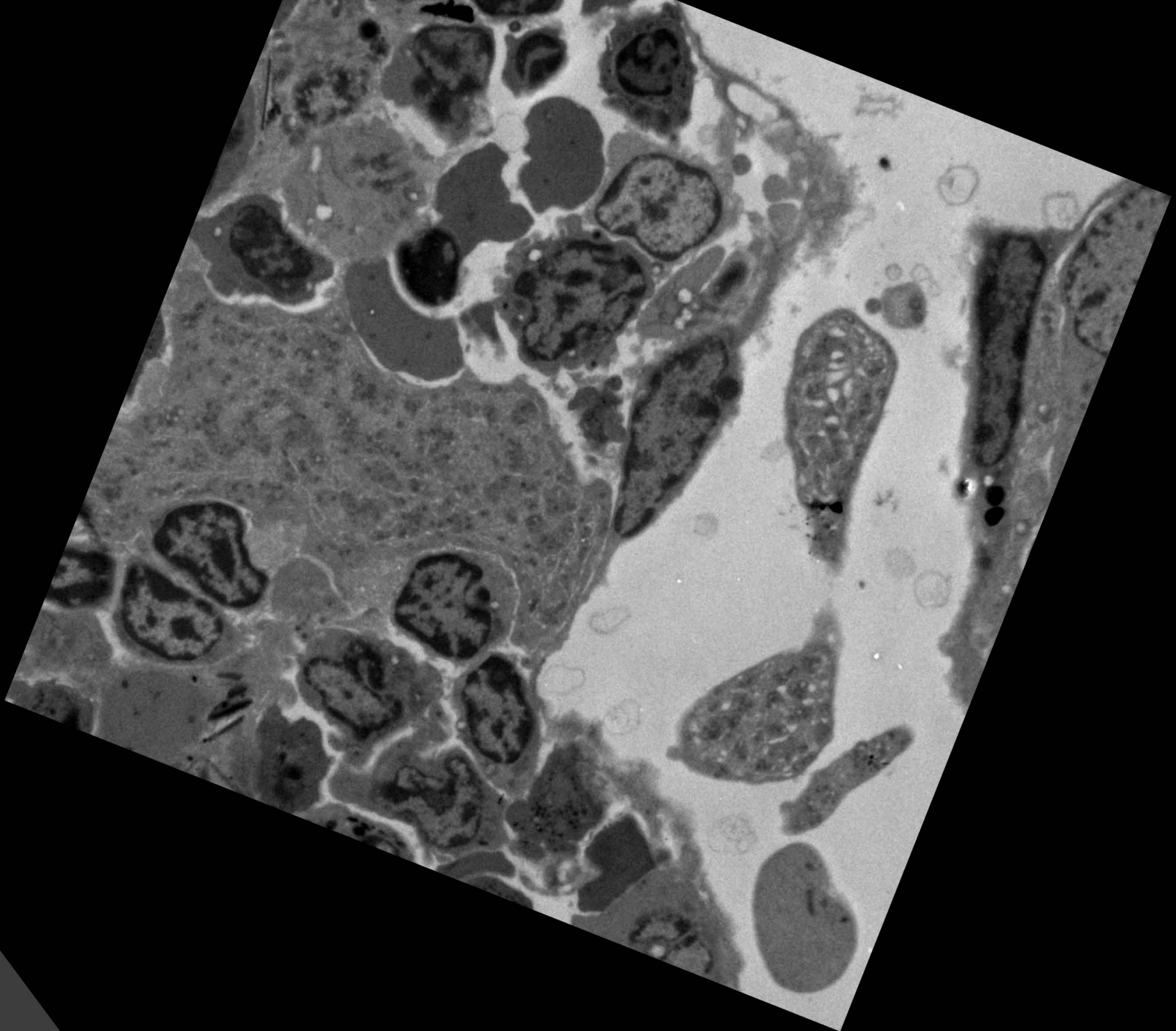

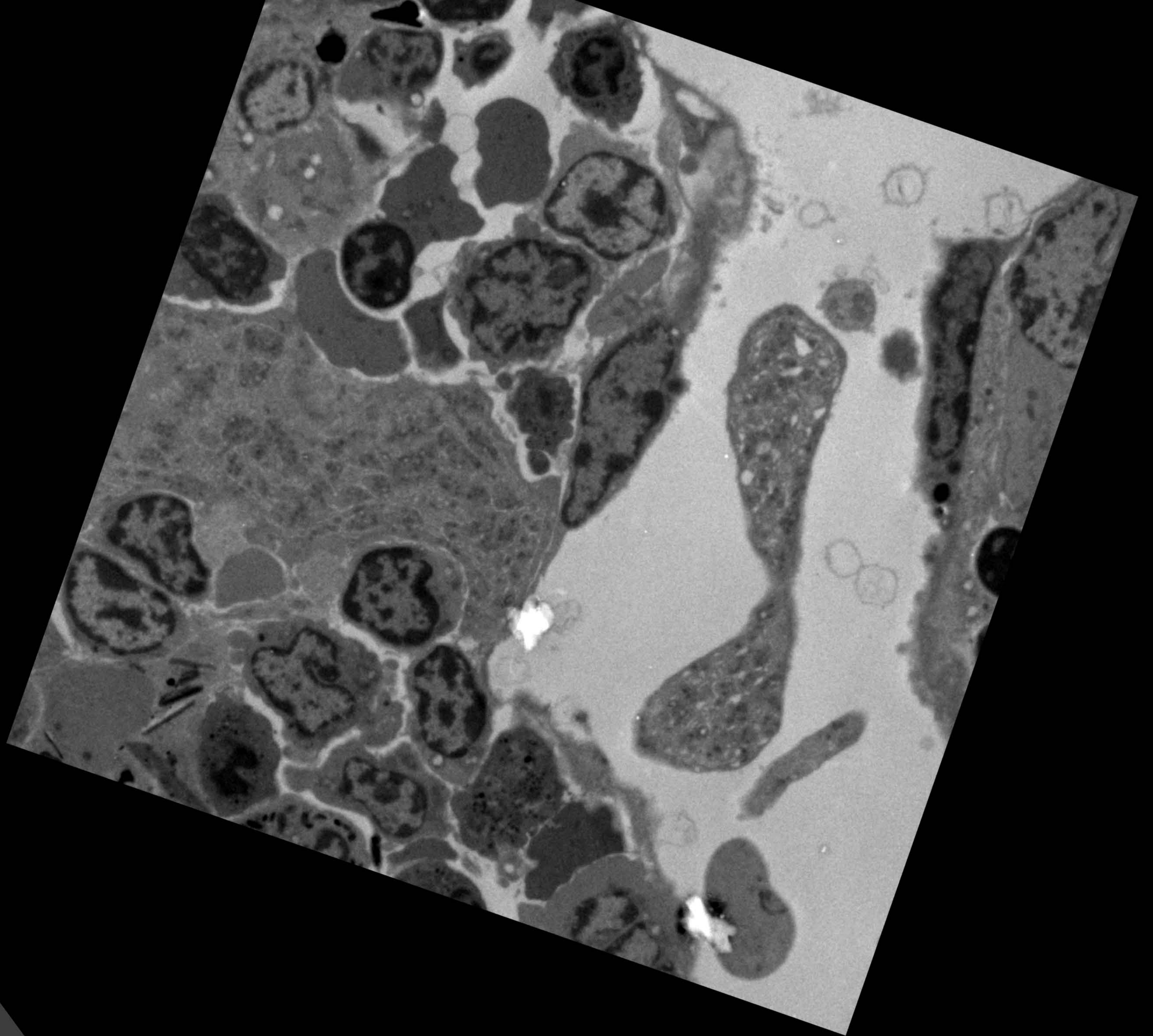

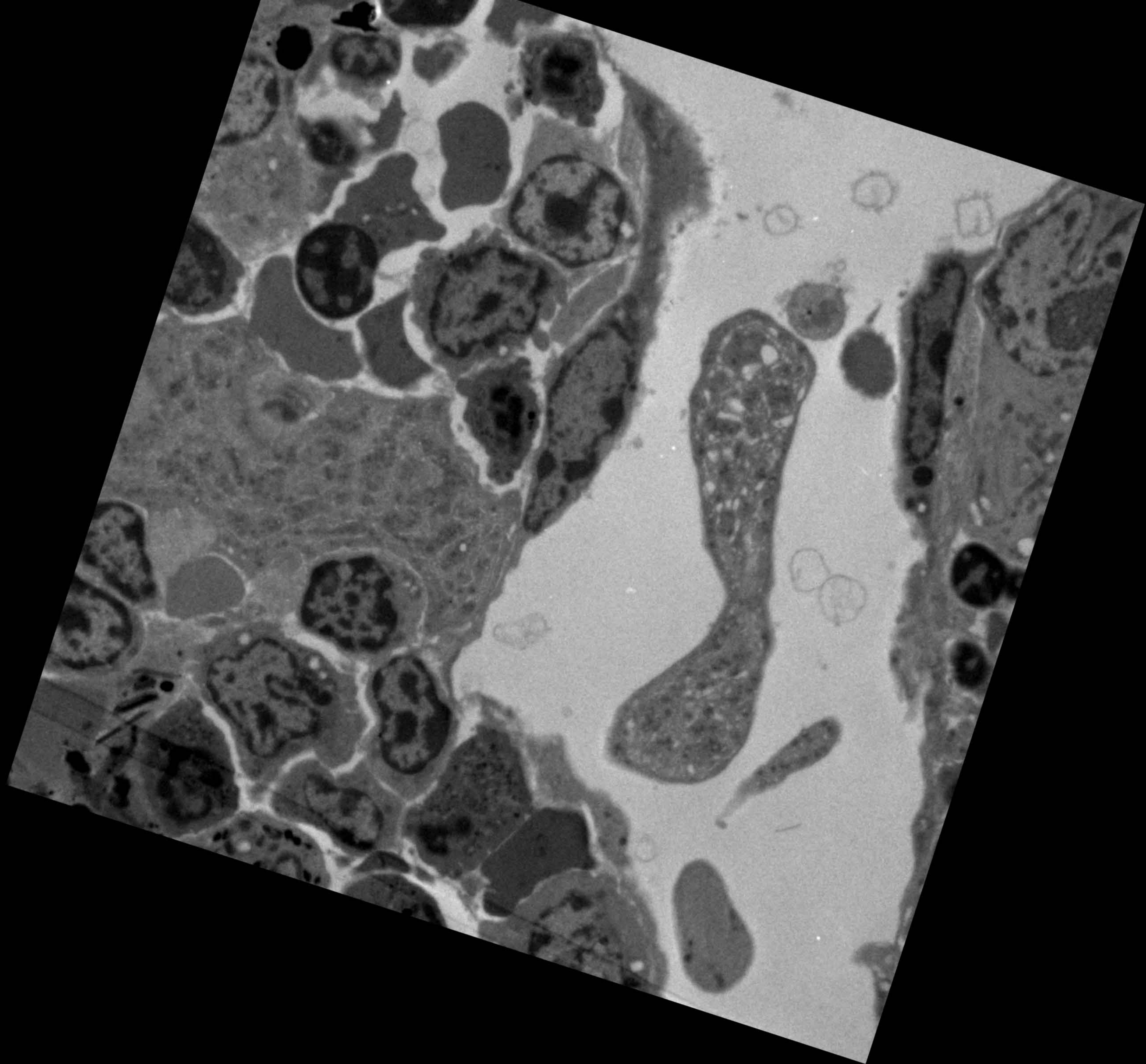

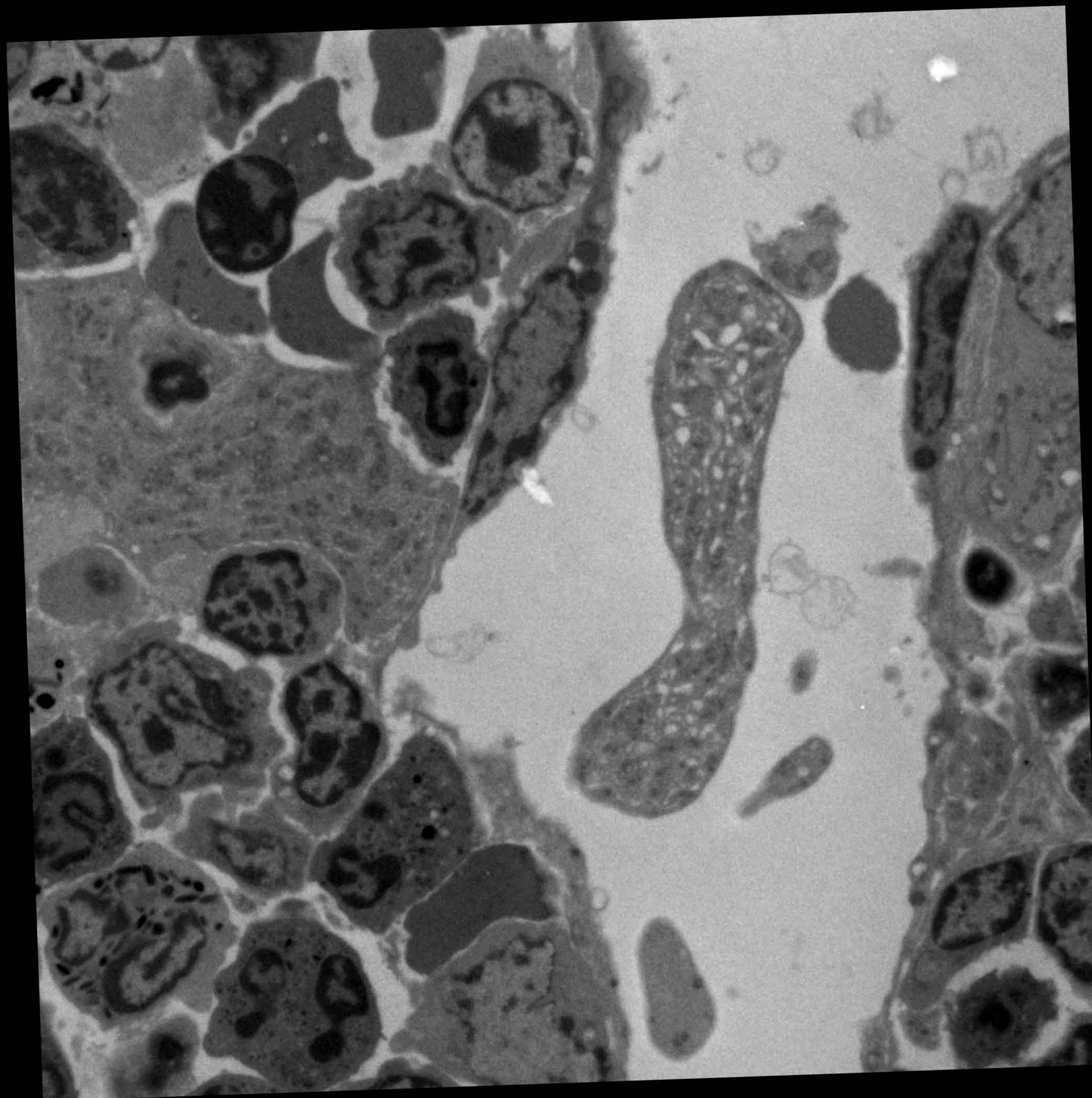

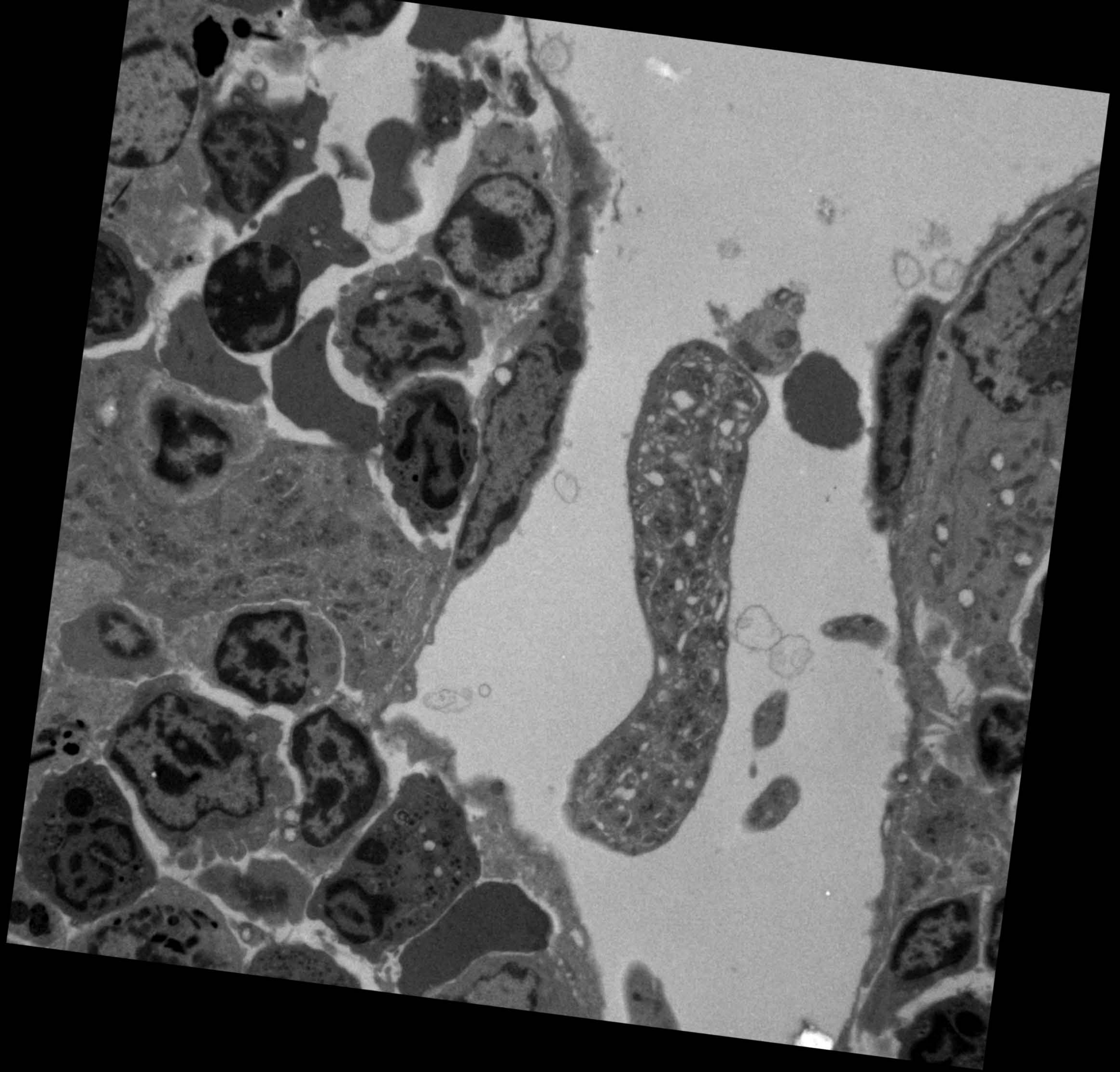

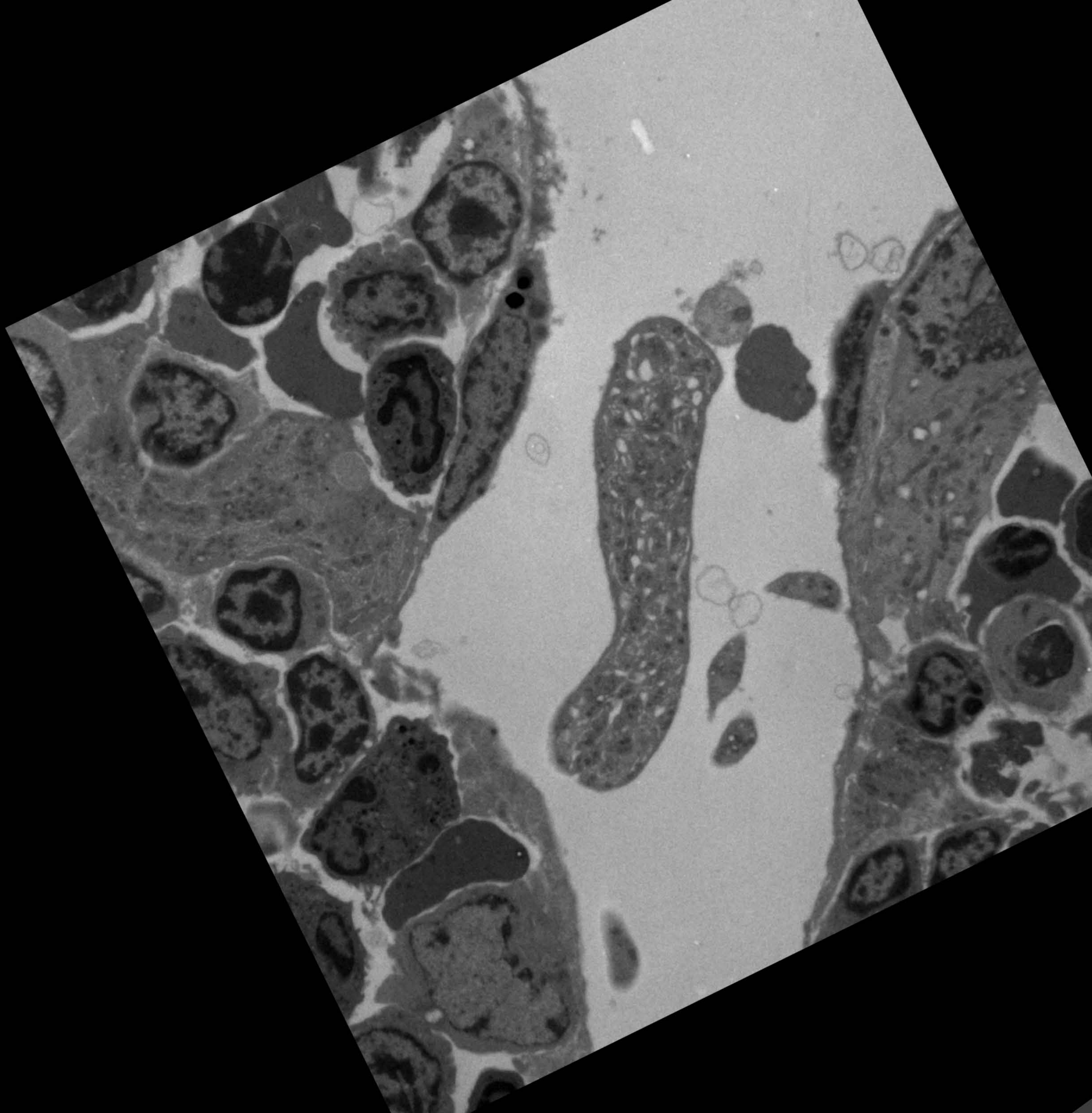

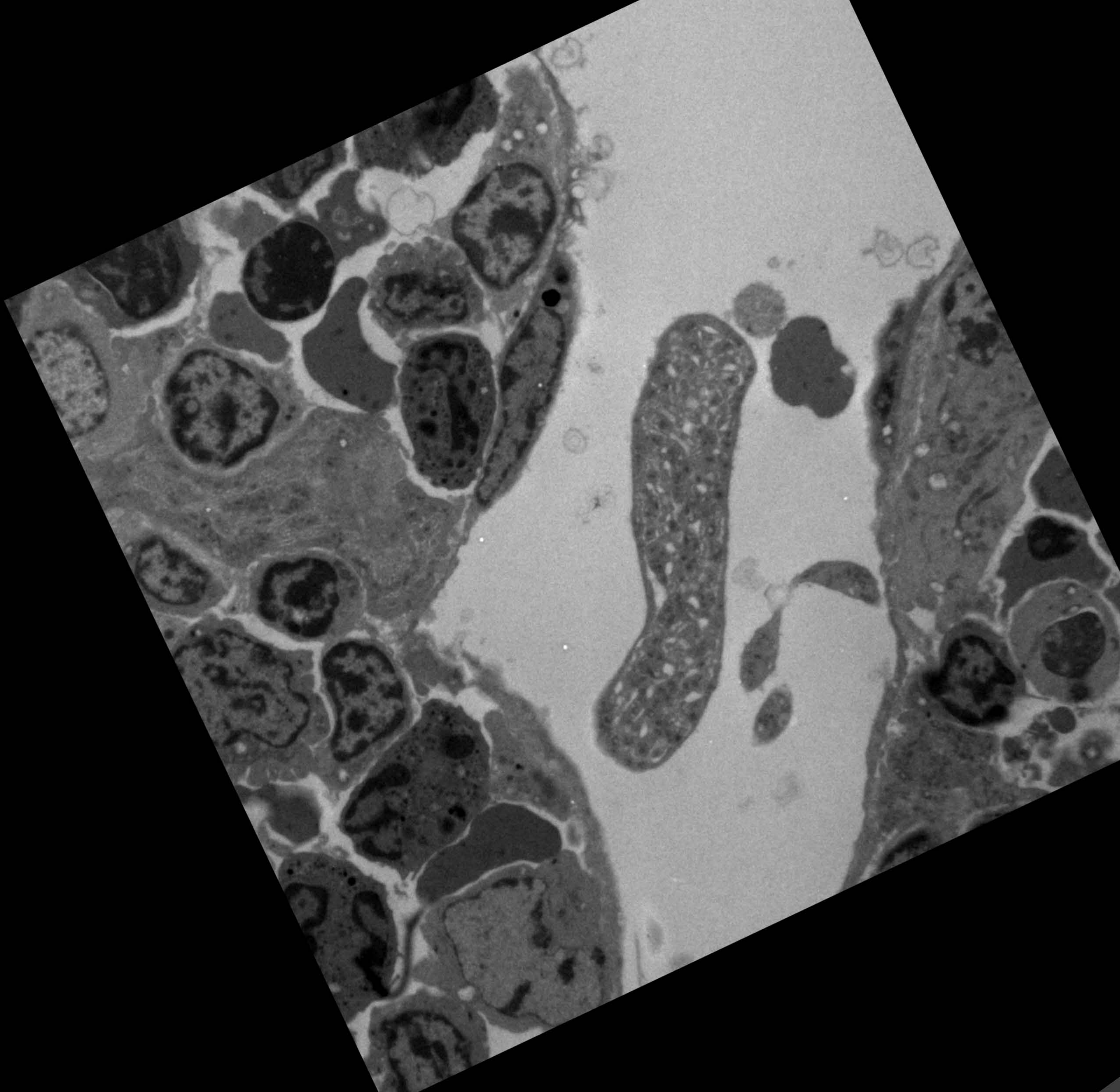

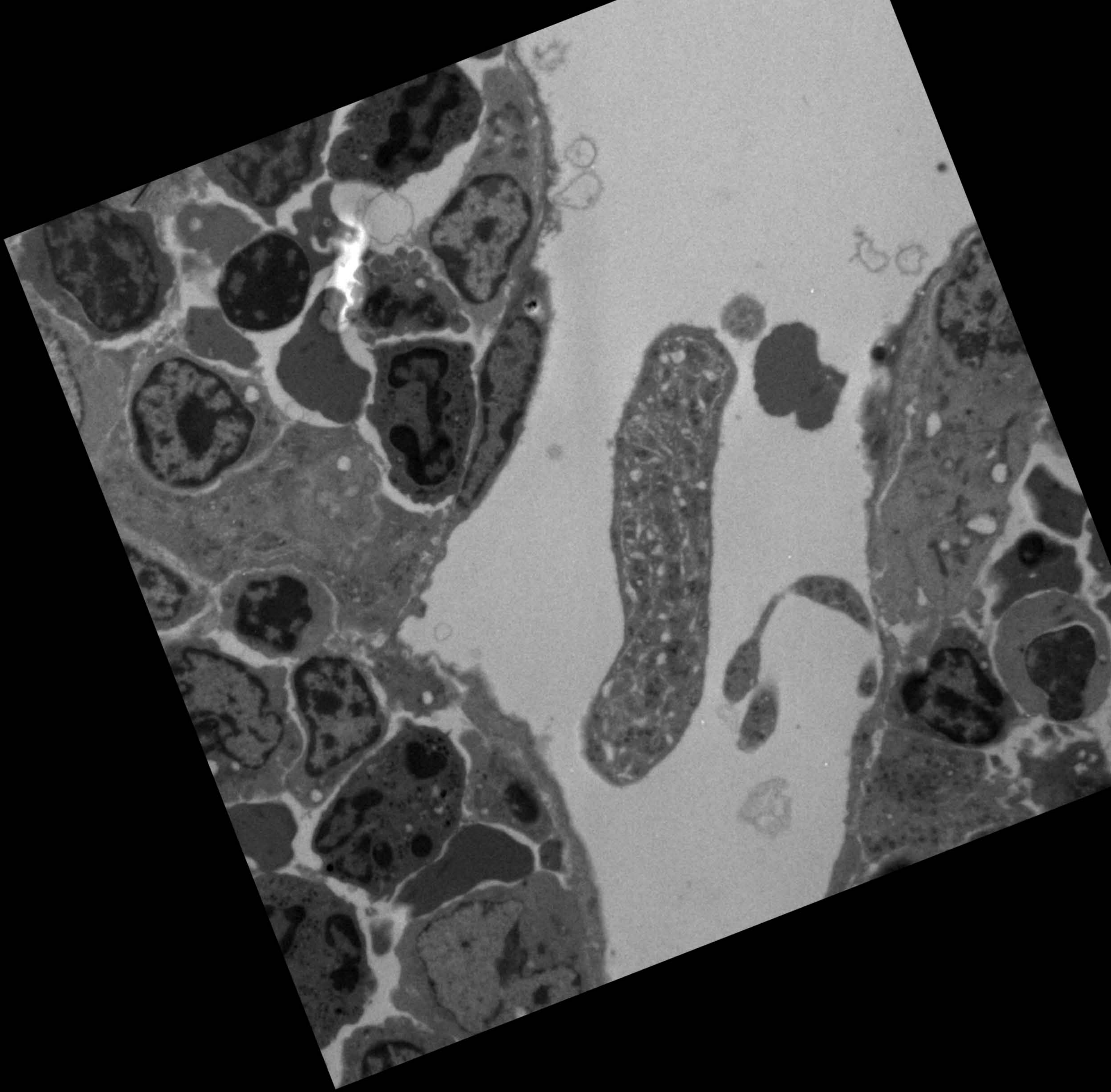

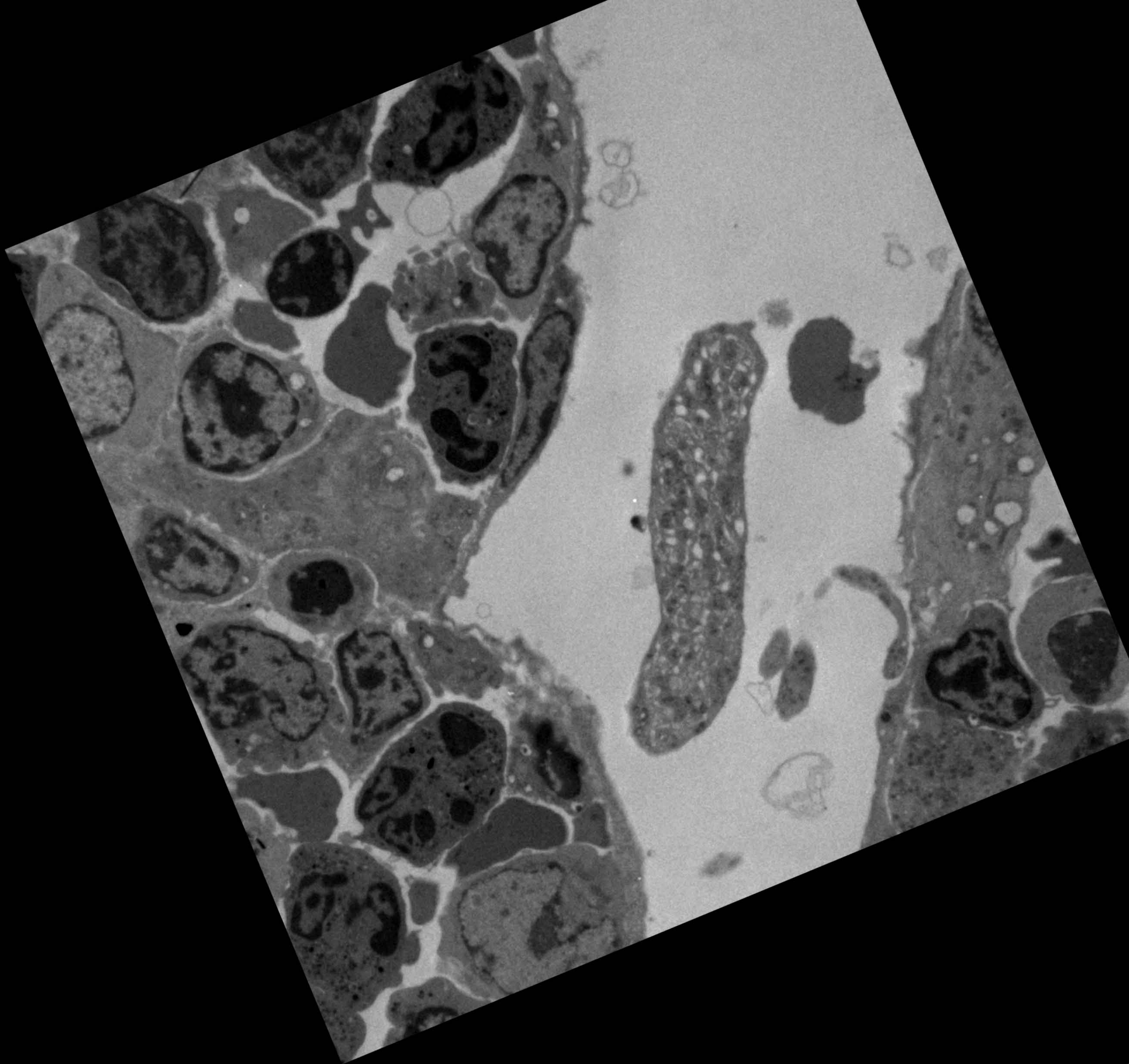

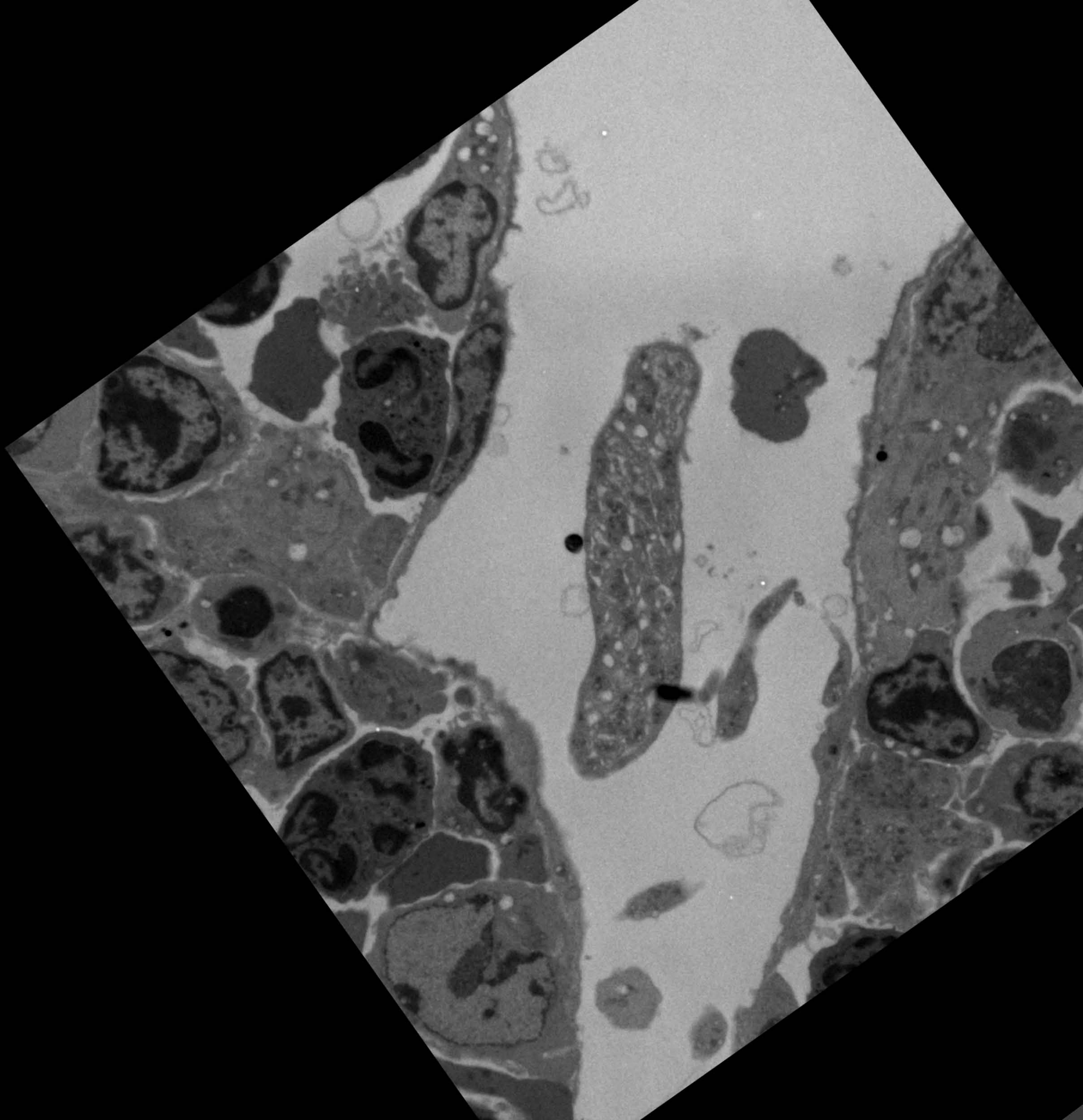

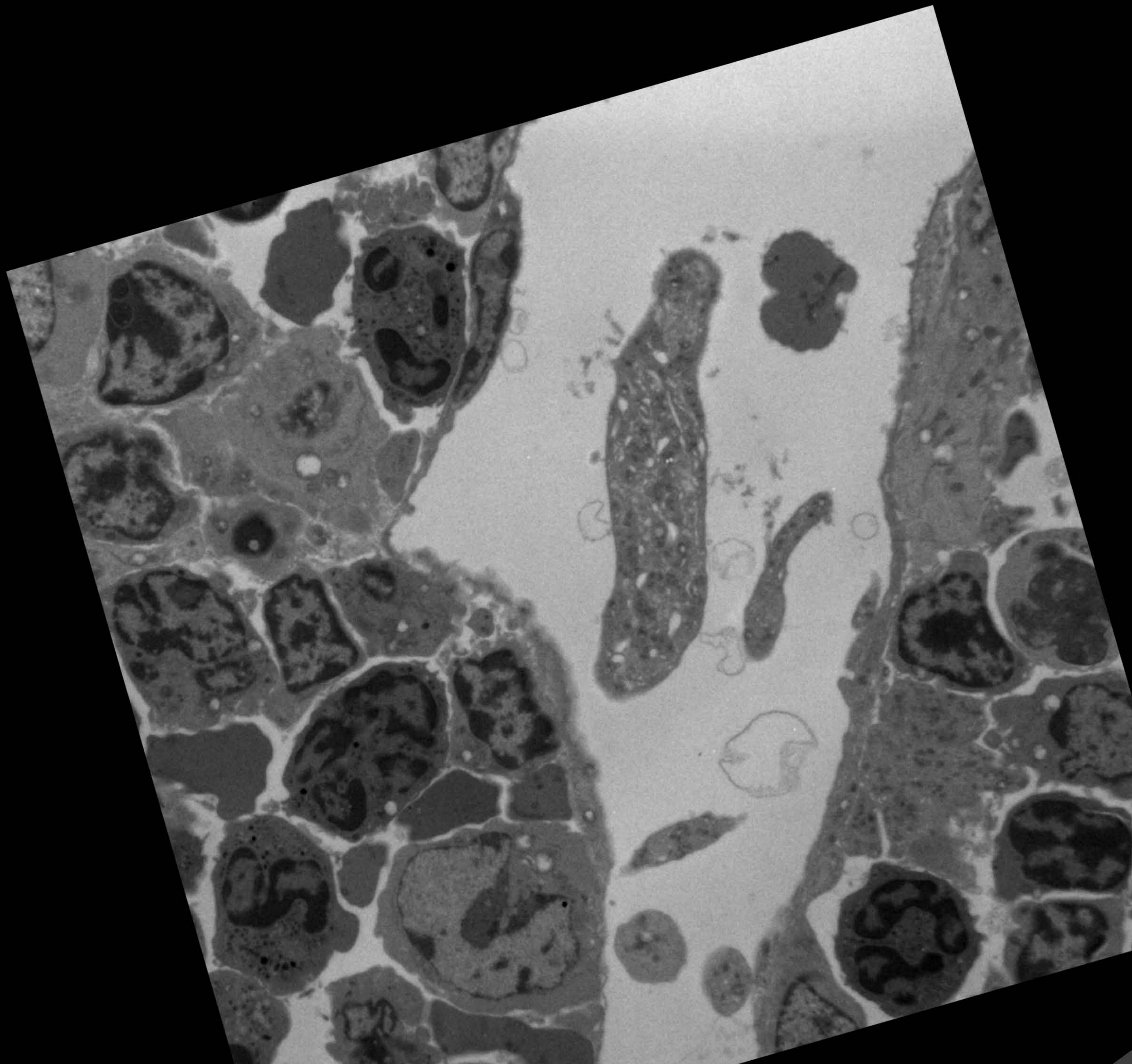

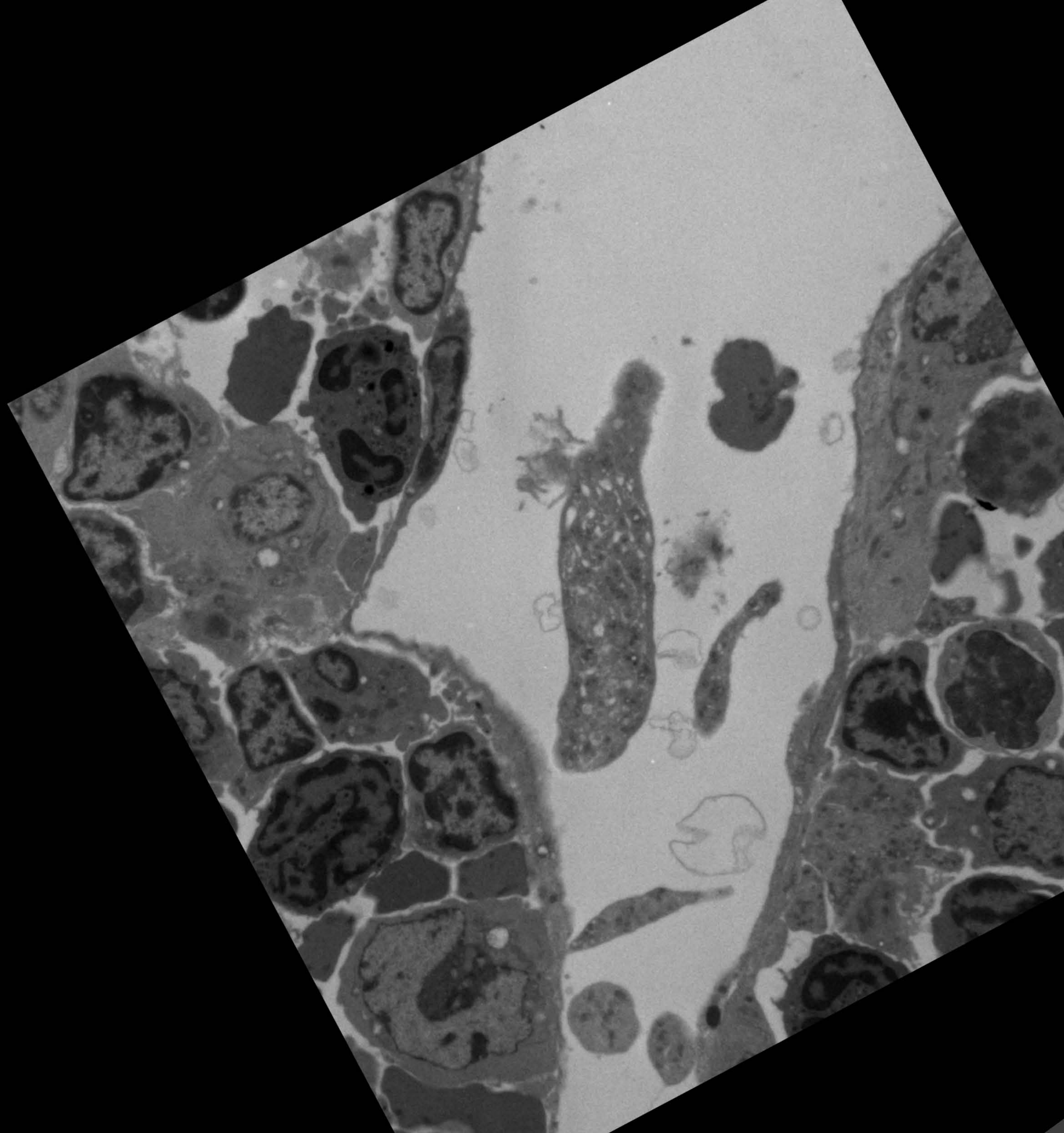

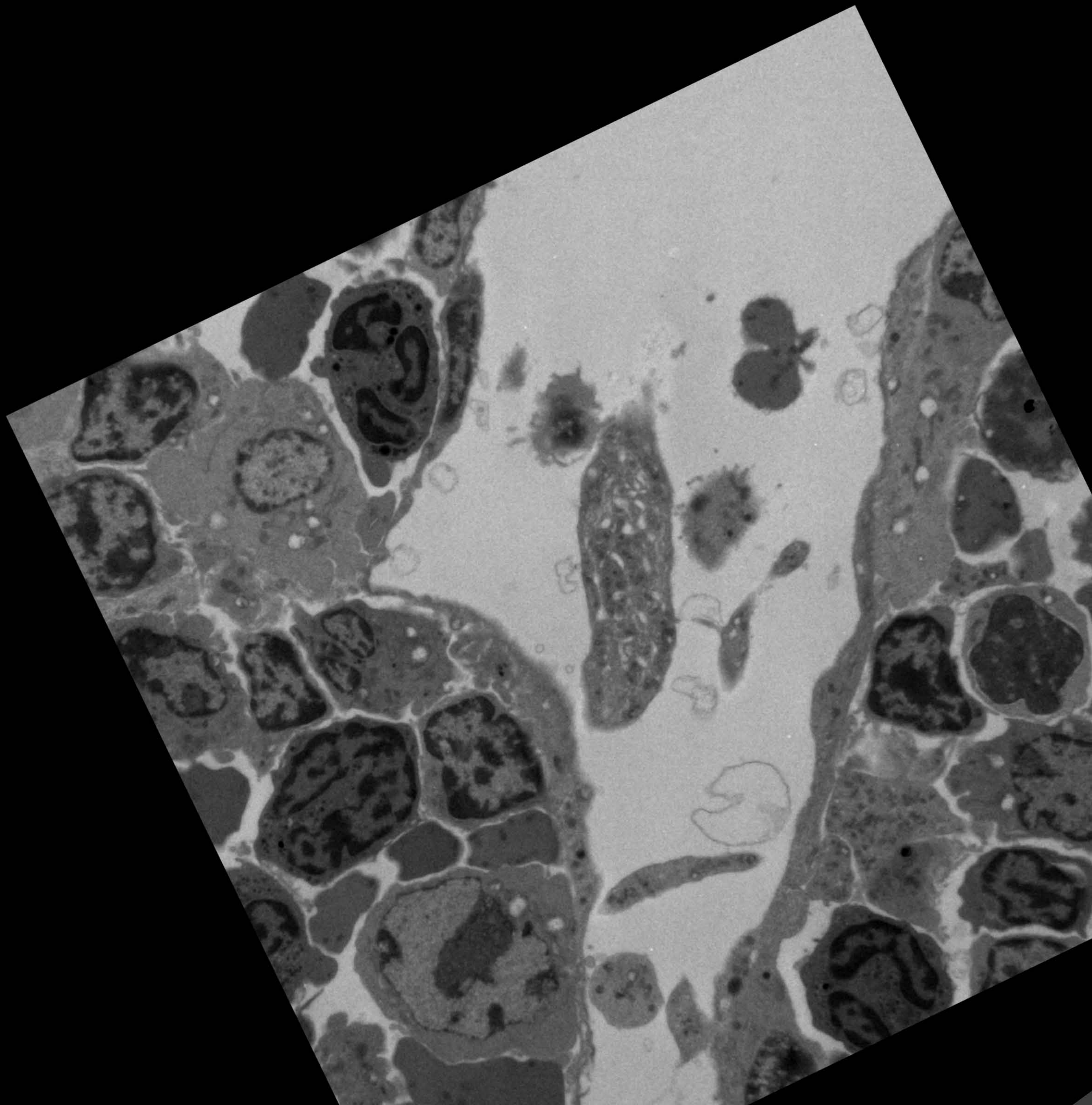

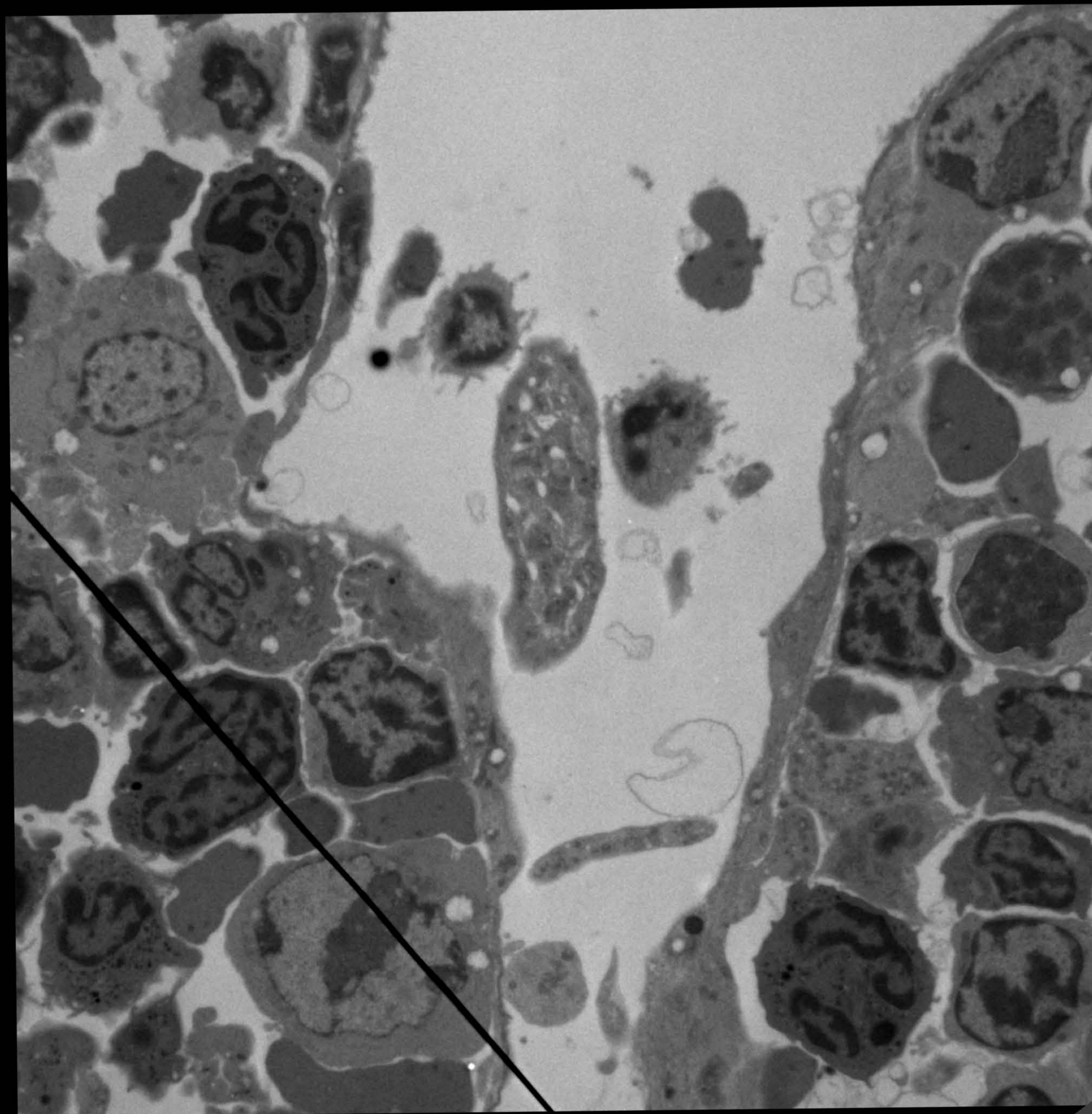

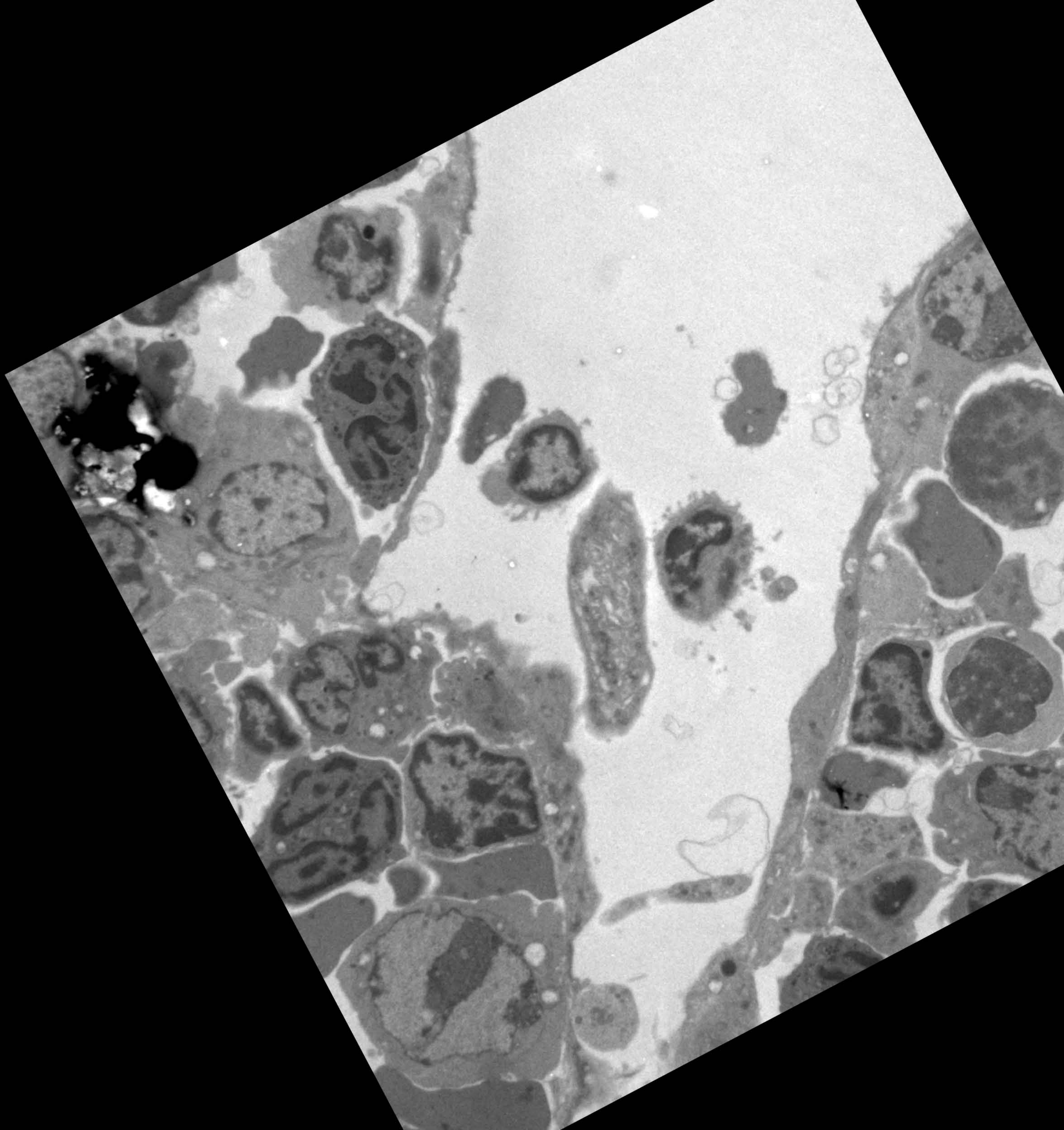

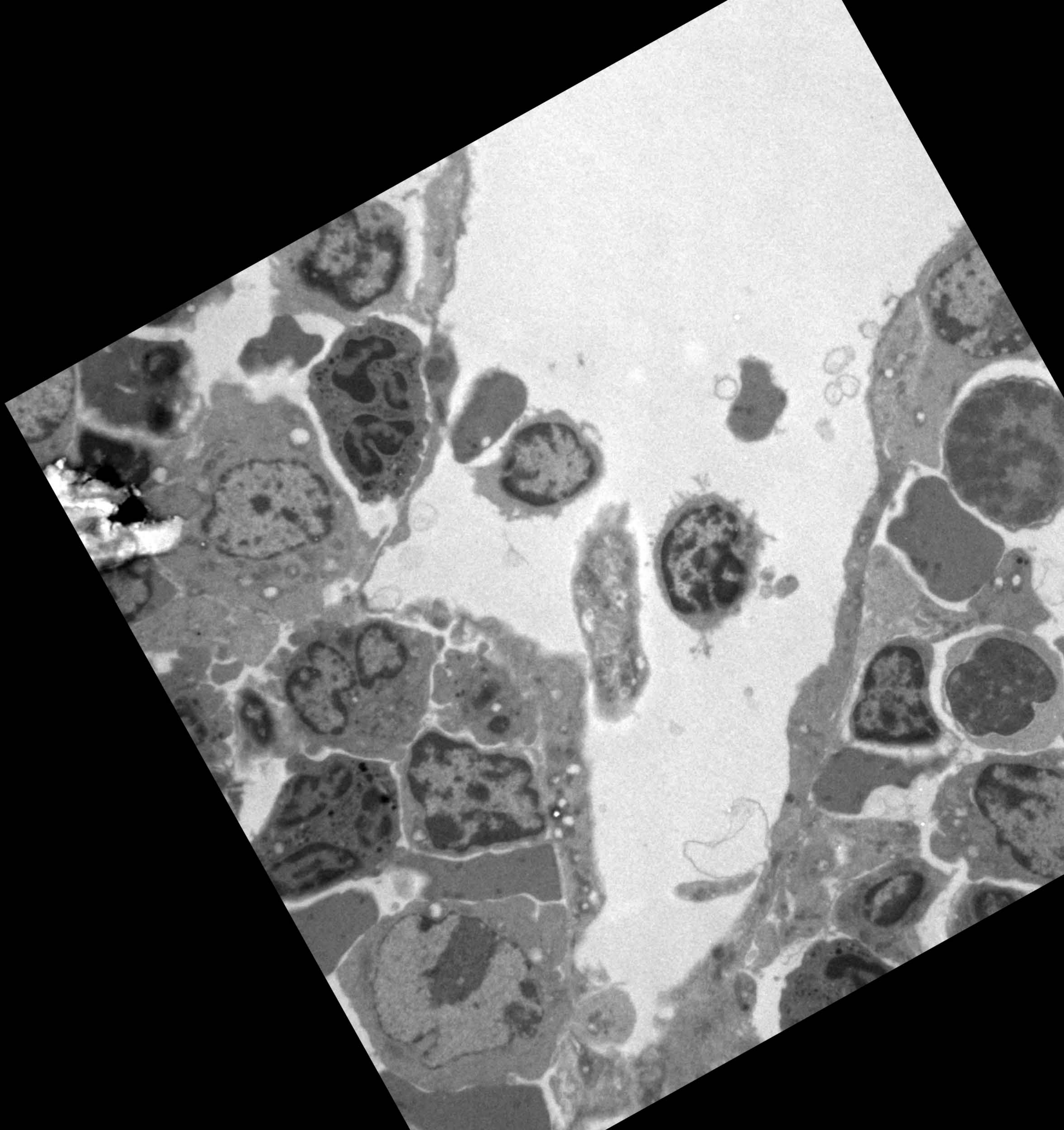

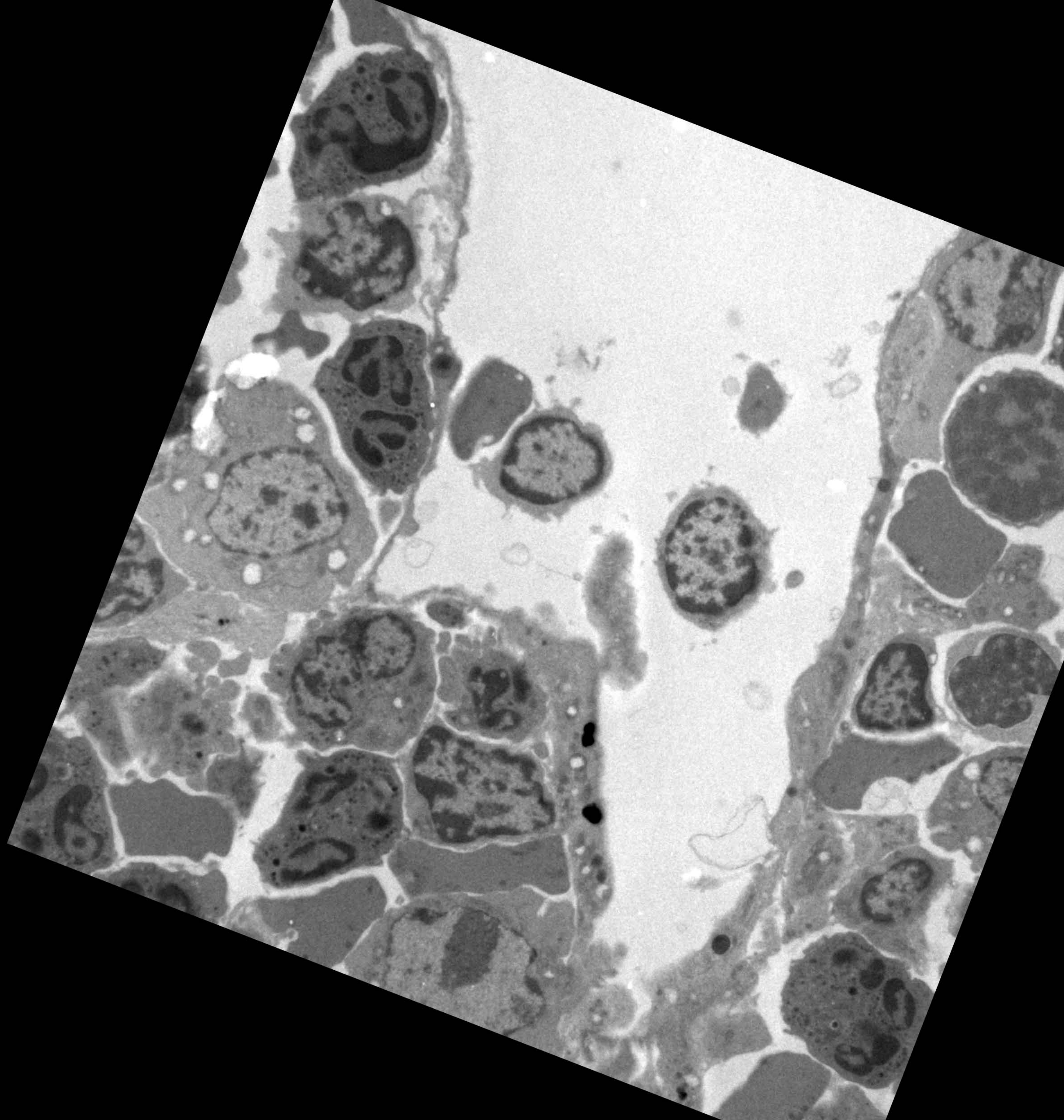

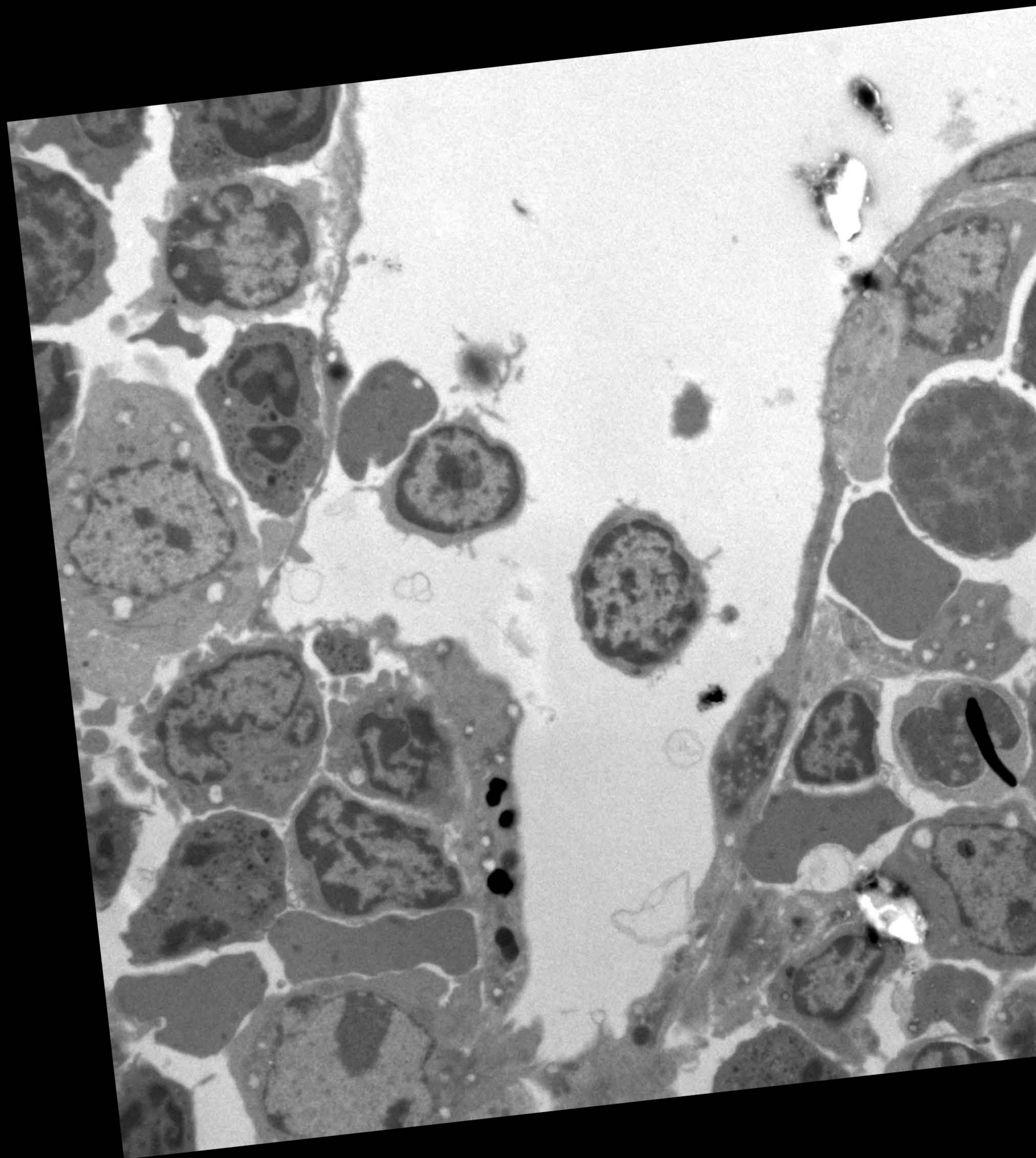

Supplement: Supplementary file 1 [file LSA-2018-00061_SdataF1.pdf]

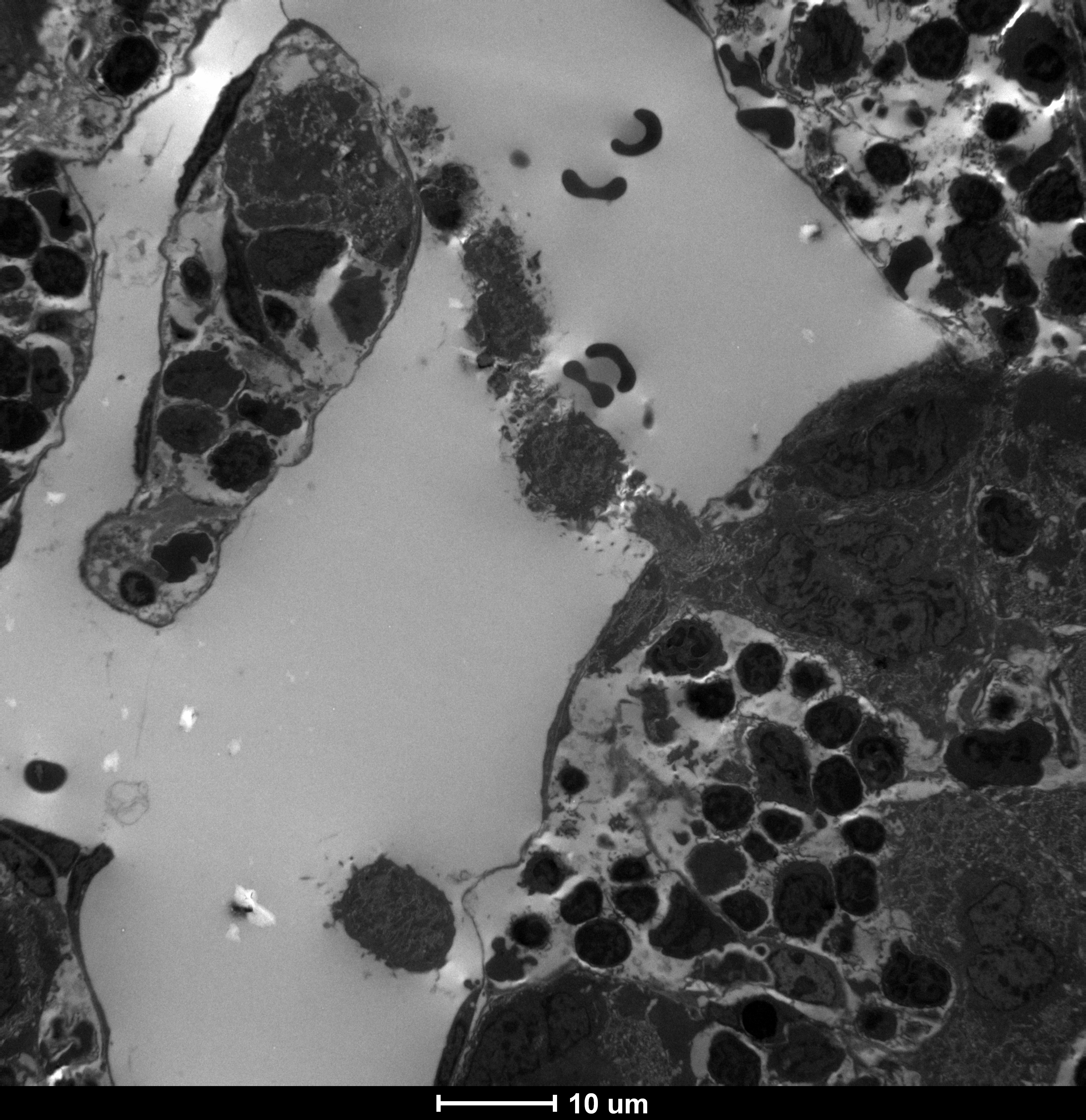

Supplement: Supplementary file 5 [file LSA-2018-00061_SdataF2.tif]

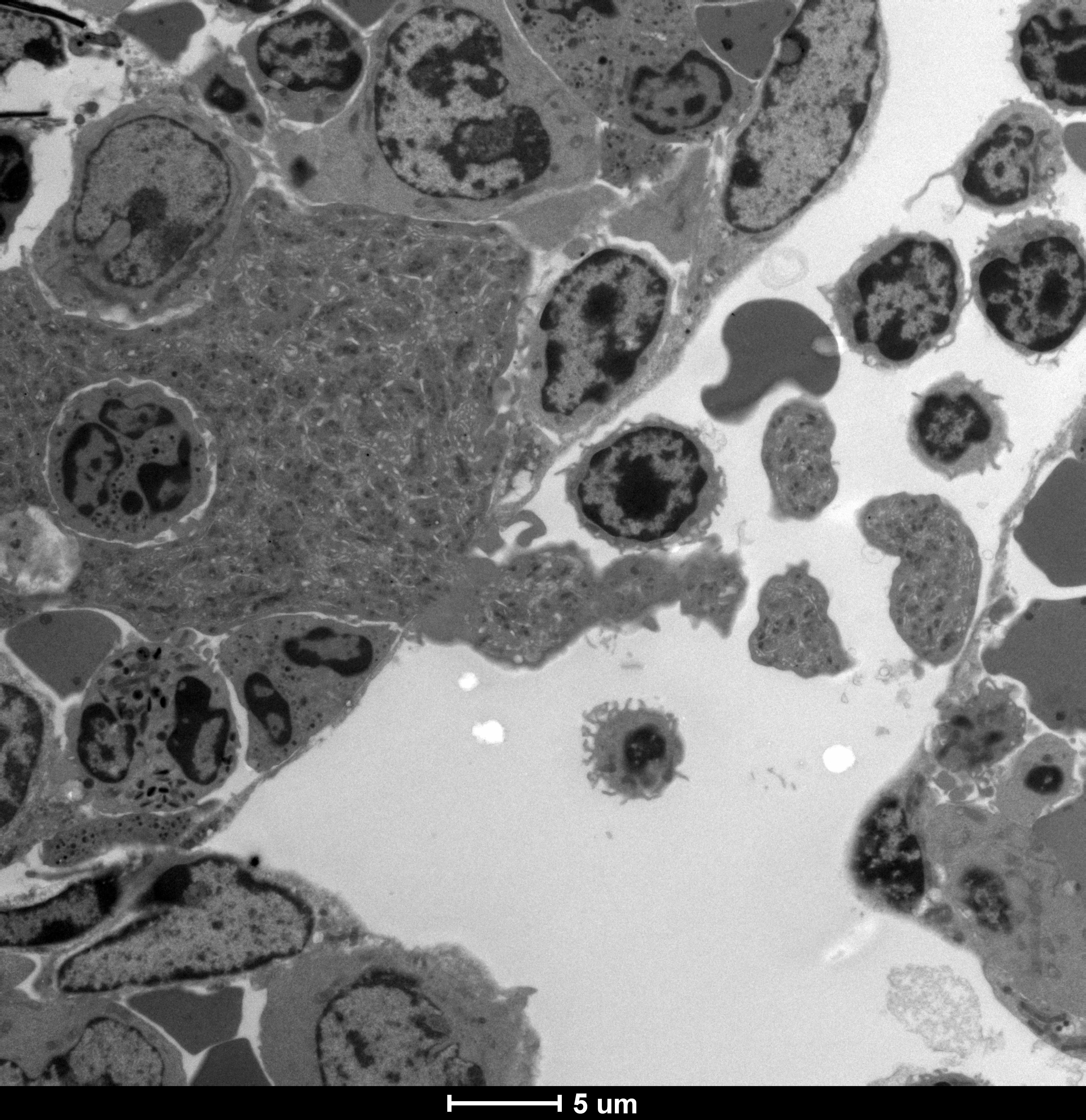

Supplement: Supplementary file 8 [file LSA-2018-00061_SdataF3.tif]

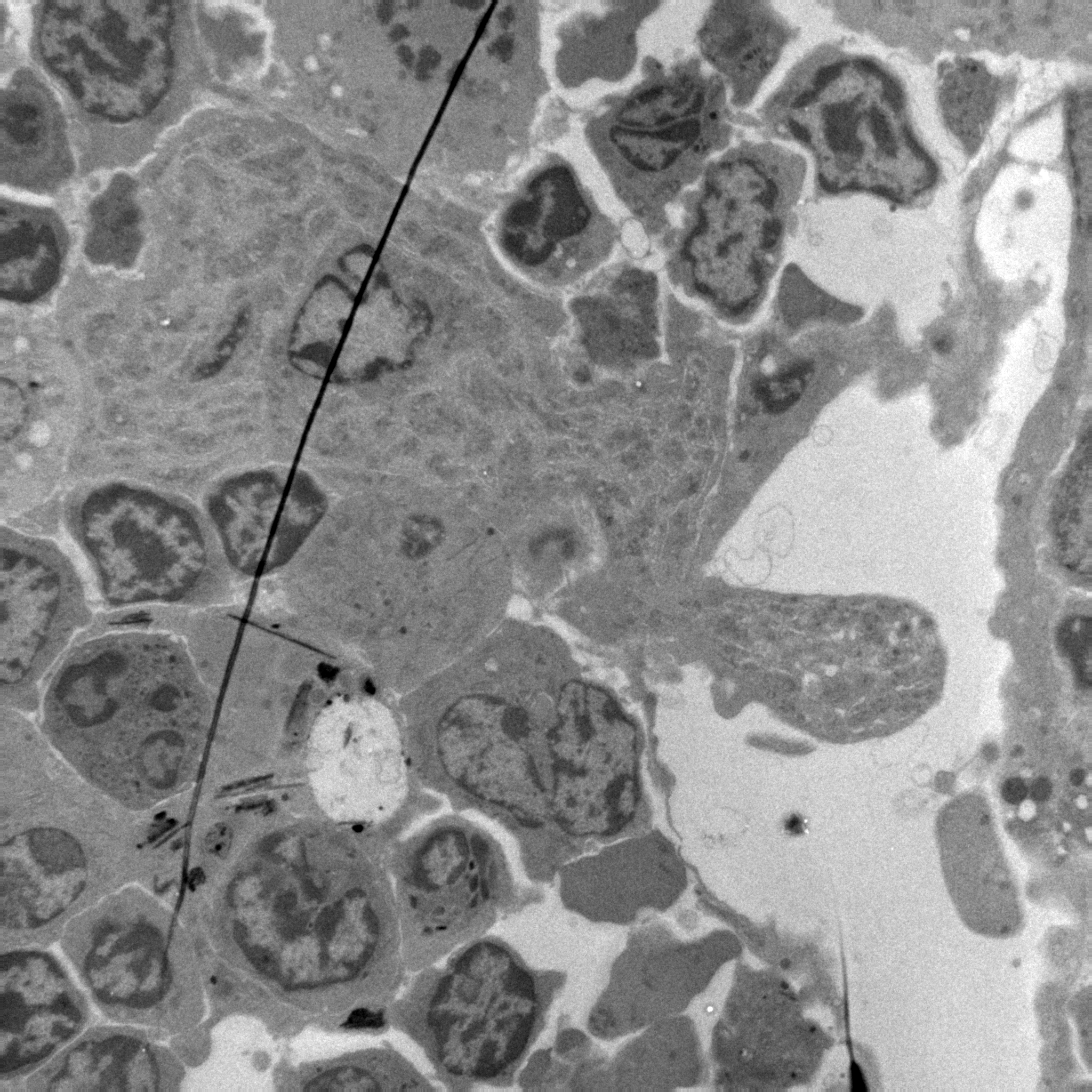

Supplement: Supplementary file 9 [file LSA-2018-00061_SdataF4.tif]

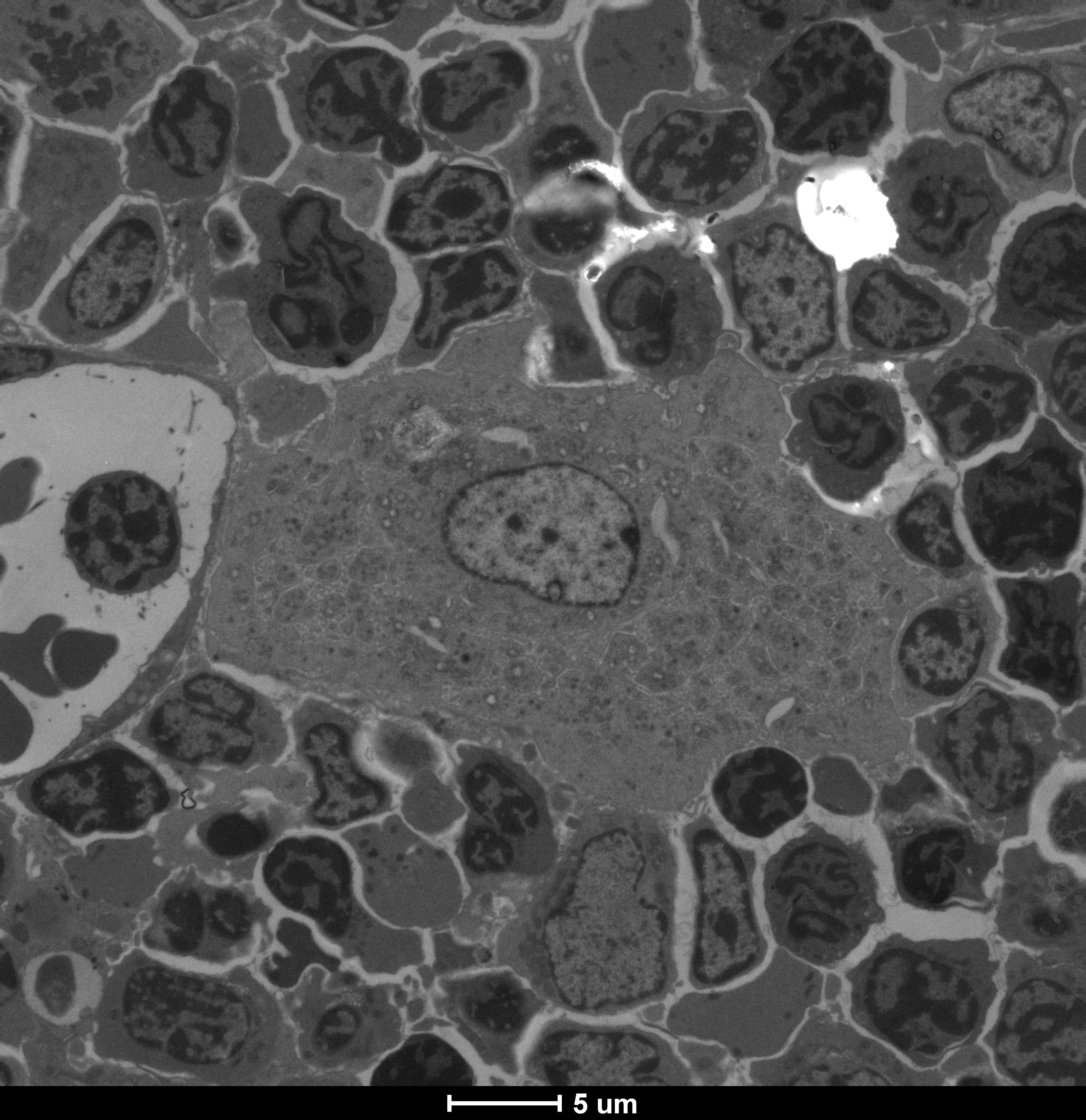

Supplement: Supplementary file 11 [file LSA-2018-00061_SdataF6.tif]

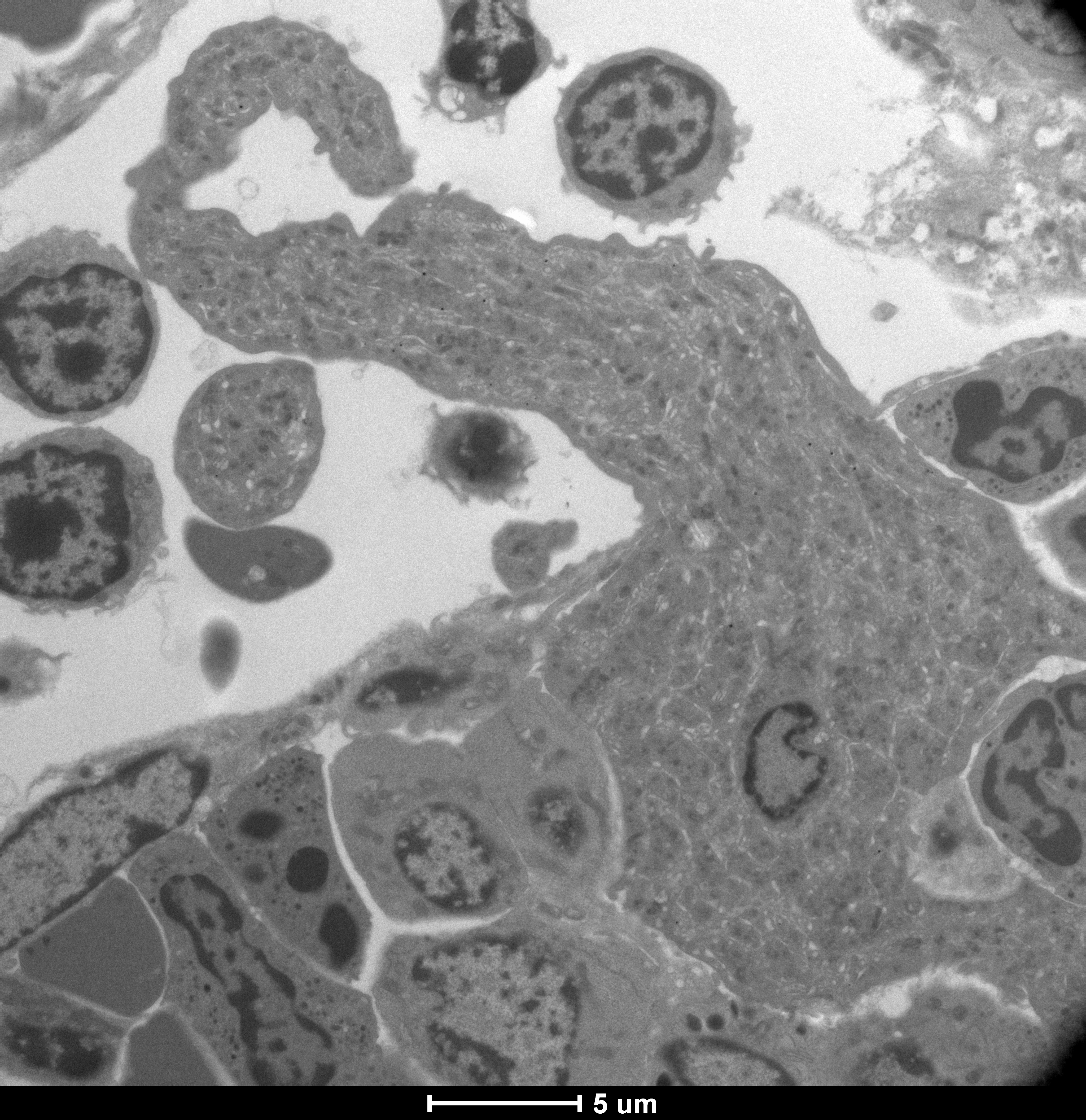

Supplement: Supplementary file 12 [file LSA-2018-00061_SdataF7.tif]
